# Dependency–competition tradeoffs structure microbial niches and nitrogen cycling

**DOI:** 10.1093/ismeco/ycag134

**Published:** 2026-05-15

**Authors:** Liang Xu, Xin Sun, Emily J Zakem

**Affiliations:** Division of Biosphere Sciences and Engineering, Carnegie Institution for Science, Pasadena, 91125 CA, United States; Department of Biology, University of Pennsylvania, Philadelphia, 19104, PA, United States; Division of Biosphere Sciences and Engineering, Carnegie Institution for Science, Pasadena, 91125 CA, United States

**Keywords:** metabolic dependencies, denitrification, trait-based approach, resource consumer model, microbial ecology, marine biogeochemistry

## Abstract

The marine nitrogen cycle is regulated by ecological interactions among diverse microbial populations. In anoxic zones, populations carrying out anaerobic metabolisms, mainly multi-step denitrification and anammox, drive the loss of bioavailable nitrogen, some of which is emitted as the potent greenhouse gas nitrous oxide (${\mathrm{N}}_2\mathrm{O}$). While competition for limiting resources is well studied, the combined effects of competition and dependencies, where a “feeder” population supplies a required resource to a “recipient,” remain poorly understood. Here, we develop a trait-based consumer–resource framework to test how recipient populations reshape the ecological niches of their feeders and competitors. Our analysis demonstrates how recipients may expand either their feeder’s or their feeder’s competitor’s niche, depending on relative competitive abilities on limiting resources. We analyze and identify equilibrium co-existence regions, threshold regimes, and the dominant pathways of nitrogen loss as a function of varying both organic matter (OM) and nitrate supply, rather than just their ratio. Examining this 2D supply space identifies a distinct zone where OM and nitrate co-limitation results in ${\mathrm{N}}_2\mathrm{O}$ production but not consumption, and thus an ecological niche for ${\mathrm{N}}_2\mathrm{O}$ accumulation. Additionally, the model suggests that anammox bacteria occupy a wider range of OM and nitrate supply regimes than denitrifying populations, consistent with their more frequent detection across diverse marine environments. The results link microbial interaction networks to biogeochemical fluxes relevant at global scales and extend ecological theory to multi-resource systems with nested competitive and dependent interactions.

## Introduction

Microbial interactions regulate global biogeochemical cycles [1–3]. In oxygen-deficient ocean regions, the nitrogen cycle is shaped by anaerobic microbes that transform bioavailable nitrogen into gaseous forms, mainly dinitrogen gas (${\mathrm{N}}_2$), driving ecosystem-scale nitrogen loss [4], and the greenhouse gas nitrous oxide (${\mathrm{N}}_2\mathrm{O}$), contributing to marine (${\mathrm{N}}_2\mathrm{O}$) emissions [5, 6]. Understanding these processes is essential for predicting nitrogen loss, which may also impact primary productivity.

Ecological theory explains microbial coexistences [7–9], but many models simplify complexity by focusing on a single resource, treating resources as substitutable or facilitation as symmetric mutualism [10–12]. However, unidirectional metabolic dependencies—i.e. asymmetric interactions—are common when microbes are limited by multiple non-substitutable resources or interactions are competitively nested [13, 14]. Related mechanisms appear in ecological succession models [15], where species modify environments to facilitate, inhibit, or tolerate others. However, succession emphasizes sequential replacement, whereas in microbial systems with continuous resource supply, similar structures can generate stable coexistence via metabolic dependencies and competition. How such dependencies shape community structure and ecosystem function remains poorly understood.

**Figure 1:**
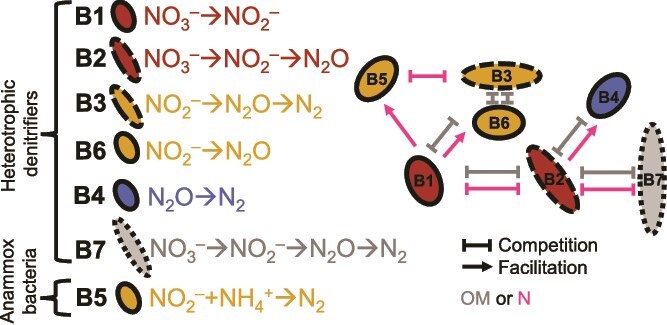
Microbial functional groups represented in the model and a schematic summary of their interactions.

Marine oxygen minimum zones (OMZs) provide natural laboratories for advancing microbial interaction theory. Here, nitrogen loss is mediated by two main functional groups: heterotrophic denitrifying microorganisms (“denitrifiers”) and anaerobic ammonium-oxidizing (anammox) bacteria [16]. Phylogenetically and functionally diverse denitrifiers use nitrate (${\mathrm{NO}}_3^{-}$) or nitrite (${\mathrm{NO}}_2^{-}$) as electron acceptors to oxidize organic matter (OM), producing intermediates along the step-wise denitrification pathway (${\mathrm{NO}}_3^{-}$  $\to$  ${\mathrm{NO}}_2^{-}$  $\to$  ${\mathrm{N}}_2\mathrm{O}$  $\to$  $\mathrm{NO}\to$  ${\mathrm{N}}_2$), often completing only part of the pathway (Fig. 1) [17, 18]. In contrast, anammox bacteria are phylogenetically conserved autotrophs that oxidize ammonia (${\mathrm{NH}}_4^{+}$) with ${\mathrm{NO}}_2^{-}$, producing ${\mathrm{N}}_2$ but not ${\mathrm{N}}_2\mathrm{O}$. These groups are metabolically interconnected, with N intermediates produced by some denitrifiers (feeders) supplying other denitrifiers and anammox bacteria (recipients). Consequently, shifts in abundance or traits, set by nutrient supplies, can restructure coexistence and alter nitrogen loss.

In this study, we ask how recipient populations modify coexistences among feeders and competitors, and how these changes affect nitrogen loss. Despite active aerobic metabolisms [19], to simplify the analysis, we focus on the dominant anaerobic N-cycling metabolisms in anoxic zones. We analyze a mechanistic consumer–resource model where microbial functional types are parameterized, in part, by the redox reactions underlying their metabolism [20]. Using invasion analysis and graphical theory, we link ecological dynamics to the 2D of OM and ${\mathrm{NO}}_3^{-}$ supply, the primary sources for elements and electrons. We analyze simplified subsets as well as the full system to demonstrate how recipients modify consumption vectors in resource ratio space, contracting or expanding feeder niches, and identify new stable regimes of nitrogen loss.

## Materials and methods

### Functional groups

We consider seven microbial functional groups representing major anaerobic nitrogen-cycling pathways in oxygen-deficient marine systems [17, 18, 21]: six denitrifier modules and anammox bacteria. Each group is defined by its essential resource requirements—OM or ${\mathrm{NH}}_4^{+}$ as an electron donor and an inorganic nitrogen species as an electron acceptor—and by its metabolic byproducts. These byproducts serve as substrates for other groups, establishing a network of competitive and unidirectional dependencies. We present these types in the order of their dependence on and competition with what we deem the two primary denitrifiers, the ${\mathrm{NO}}_3^{-}\to{\mathrm{NO}}_2^{-}$ denitrifier and the ${\mathrm{N}\mathrm{O}}_3^{-}\to{\mathrm{N}}_2\mathrm{O}$ denitrifier (Fig. 1):

1.
${\mathrm{NO}}_3^{-}\to{\mathrm{NO}}_2^{-}$ denitrifiers (${\mathrm{B}}_1$): heterotrophs that use OM and ${\mathrm{NO}}_3^{-}$ to produce ${\mathrm{NO}}_2^{-}$.
2.
${\mathrm{N}\mathrm{O}}_3^{-}\to{\mathrm{N}}_2\mathrm{O}$ denitrifiers (${\mathrm{B}}_2$): heterotrophs that use OM and ${\mathrm{NO}}_3^{-}$ to produce ${\mathrm{N}}_2\mathrm{O}$.
3.
${\mathrm{N}\mathrm{O}}_2^{-}\to{\mathrm{N}}_2$ denitrifiers (${\mathrm{B}}_3$): heterotrophs requiring OM and ${\mathrm{NO}}_2^{-}$, producing ${\mathrm{N}}_2$.
4.
${\mathrm{N}}_2\mathrm{O}\to{\mathrm{N}}_2$ denitrifiers (${\mathrm{B}}_4$): heterotrophs requiring OM and ${\mathrm{N}}_2\mathrm{O}$, producing ${\mathrm{N}}_2$.
5. Anammox bacteria (${\mathrm{B}}_5$): hutotrophs oxidizing ${\mathrm{NH}}_4^{+}$ with ${\mathrm{NO}}_2^{-}$, producing ${\mathrm{N}}_2$. Although some ${\mathrm{NO}}_3^{-}$ is released [22], this fraction is small and is neglected for simplicity.
6.
${\mathrm{N}\mathrm{O}}_2^{-}\to{\mathrm{N}}_2\mathrm{O}$ denitrifiers (${\mathrm{B}}_6$): Heterotrophs requiring OM and ${\mathrm{NO}}_2^{-}$, producing ${\mathrm{N}}_2\mathrm{O}$.
7. Complete denitrifiers (${\mathrm{B}}_7$): Heterotrophs that reduce ${\mathrm{NO}}_3^{-}$ all the way to ${\mathrm{N}}_2$, using OM as the electron donor.

### Model framework

To formalize these interactions, we implement a virtual chemostat-based consumer–resource model tracking microbial biomasses and the concentrations of OM, ${\mathrm{NO}}_3^{-}$, ${\mathrm{NO}}_2^{-}$, ${\mathrm{N}}_2\mathrm{O}$, and ${\mathrm{NH}}_4^{+}$. Growth of each functional group depends on two essential and non-substitutable resources (an electron donor and an electron acceptor). The model links the physiological traits of each group—maximum uptake rates, half-saturation constants, and biomass yields—to their realized growth rates and subsistence concentrations as described below. This structure enables a direct comparison of competitive abilities while dependency links emerge from excretions.

The dynamics are generally given by:


(1) \begin{eqnarray*} \frac{1}{B_i}\frac{d{B}_i}{dt}={\mu}_i-a \end{eqnarray*}

(2) \begin{eqnarray*} \frac{d{R}_j}{dt}=a\left({s}_j-{R}_j\right)+\sum_{i=1}^7{E}_{ij}{B}_i-\sum_{i=1}^7{V}_{ij}^r{B}_i. \end{eqnarray*}

Here, ${B}_i$ denotes the biomass of microbial group $i$, and ${R}_j$ the concentration of resource $j$. The parameter $a$ is the dilution rate, ${\mu}_i$ is the realized growth rate of group $i$, and ${V}_{ij}^r$ represents the uptake rate of resource $j$ by group $i$ (see below for details). The term ${s}_j$ denotes the external supply rate of resource $j$, while ${E}_{ij}$ is the excretion matrix specifying which resources are released by each group.

We compute growth and uptake, using Liebig’s Law of the minimum, as follows. For each essential resource, we first calculate the potential growth rate it can support. The realized uptake rate ${V}_{ij}^r$ is set by the maximum specific uptake rate ${V}_{ij}^m$ and the half saturation constant ${K}_{ij}$, and these uptake kinetic parameters are the same for each substrate for the competing denitrifier types (Table 1; as in [20]). The potential growth rate is then:


(3) \begin{eqnarray*} {\mu}_{ij}={A}_{ij}\;{y}_{ij}\;{V}_{ij}^m\;\frac{R_j}{R_j+{K}_{ij}}, \end{eqnarray*}

where ${y}_{ij}$ is the biomass yield (mol *B* synthesized per mol *R* utilized) and ${A}_{ij}$ indicates whether group $i$ uses resource $j$, given by


(4) \begin{eqnarray*} {\displaystyle \begin{array}{@{}l@{}}\mathrm{O}\mathrm{M}\kern0.5em {\mathrm{N}\mathrm{O}}_3^{-}\kern0.5em {\mathrm{N}\mathrm{O}}_2^{-}\kern0.5em {\mathrm{N}}_2\mathrm{O}\kern0.5em {\mathrm{N}\mathrm{H}}_4^{+}\\{}{A}_{ij}=\left(\begin{array}{@{}ccccc@{}}1& 1& 0& 0& 0\\{}1& 1& 0& 0& 0\\{}1& 0& 1& 0& 0\\{}1& 0& 0& 1& 0\\{}0& 0& 1& 0& 1\\{}1& 0& 1& 0& 0\\{}1& 1& 0& 0& 0\end{array}\right)\begin{array}{@{}l@{}}{B}_1:{\mathrm{N}\mathrm{O}}_3^{-}\to{\mathrm{N}\mathrm{O}}_2^{-}\\{}{B}_2:{\mathrm{N}\mathrm{O}}_3^{-}\to{\mathrm{N}}_2\mathrm{O}\\{}{B}_3:{\mathrm{N}\mathrm{O}}_2^{-}\to{\mathrm{N}}_2\mathrm{O}\to{\mathrm{N}}_2\\{}{B}_4:{\mathrm{N}}_2\mathrm{O}\to{\mathrm{N}}_2\\{}{B}_5:{\mathrm{N}\mathrm{O}}_2^{-}+{\mathrm{N}\mathrm{H}}_4^{+}\to{\mathrm{N}}_2\\{}{B}_6:{\mathrm{N}\mathrm{O}}_2^{-}\to{\mathrm{N}}_2\mathrm{O}\\{}{B}_7:{\mathrm{N}\mathrm{O}}_3^{-}\to{\mathrm{N}\mathrm{O}}_2^{-}\to{\mathrm{N}}_2\mathrm{O}\to{\mathrm{N}}_2\end{array}\end{array}} \end{eqnarray*}

**Table 1:** Physiological trait values are identical to those used in Sun et al. [20].

Microbes	Substrate	Max uptake rate ${V}_{ij}^m$ (mol N ${d}^{-1}$)	Yield ${y}_{ij}$	Half-saturation rate ${K}_{ij}$ ($\mathrm{\mu} \mathrm{M}$ N)	${R}_{ij}^{\ast }$
${B}_1:{\mathrm{NO}}_3^{-}\rightarrow{\mathrm{NO}}_2^{-}$	${R}_1:$ OM	1.377	0.15	0.1	0.024
${B}_1:{\mathrm{NO}}_3^{-}\rightarrow{\mathrm{NO}}_2^{-}$	${R}_2:$ ${\mathrm{NO}}_3^{-}$	50.8	0.011	4	0.295
${B}_2:{\mathrm{N}\mathrm{O}}_3^{-}\rightarrow{\mathrm{N}}_2\mathrm{O}$	${R}_1:$ OM	1.377	0.148	0.1	0.0245
${B}_2:{\mathrm{N}\mathrm{O}}_3^{-}\rightarrow{\mathrm{N}}_2\mathrm{O}$	${R}_2:$ ${\mathrm{NO}}_3^{-}$	50.8	0.023	4	0.144
${B}_3:{\mathrm{N}\mathrm{O}}_2^{-}\to{\mathrm{N}}_2$	${R}_1:$ OM	1.377	0.197	0.1	0.0172
${B}_3:{\mathrm{N}\mathrm{O}}_2^{-}\to{\mathrm{N}}_2$	${R}_3:$ ${\mathrm{NO}}_2^{-}$	50.8	0.024	4	0.138
${B}_4:{\mathrm{N}}_2\mathrm{O}\to{\mathrm{N}}_2$	${R}_1:$ OM	1.377	0.298	0.1	0.0108
${B}_4:{\mathrm{N}}_2\mathrm{O}\rightarrow{\mathrm{N}}_2$	${R}_4:$ ${\mathrm{N}}_2\mathrm{O}$	50.8	0.013	0.6	0.039
${B}_5: ANAMMOX$	${R}_5:$ ${\mathrm{NH}}_4^{+}$	50.8	0.013	0.45	0.028
${B}_5: ANAMMOX$	${R}_3:$ ${\mathrm{NO}}_2^{-}$	50.8	0.011	0.45	0.034
${B}_6:{\mathrm{N}\mathrm{O}}_2^{-}\to{\mathrm{N}}_2\mathrm{O}$	${R}_1:$ OM	1.377	0.201	0.1	0.0169
${B}_6:{\mathrm{N}\mathrm{O}}_2^{-}\to{\mathrm{N}}_2\mathrm{O}$	${R}_3:$ ${\mathrm{NO}}_2^{-}$	50.8	0.016	4	0.2066
${B}_7:{\mathrm{NO}}_3^{-}\to \mathrm{N}_2$	${R}_1:$ OM	1.377	0.138	0.1	0.0268
${B}_7:{\mathrm{NO}}_3^{-}\to \mathrm{N}_2$	${R}_2:$ ${\mathrm{NO}}_3^{-}$	50.8	0.026	4	0.124

The subsistence concentration (${R}_{ij}^{\ast }$) for each resource [8] is defined as the concentration at which growth balances mortality:


(5) \begin{eqnarray*} {R}_{ij}^{\ast }=\frac{K_{ij}a}{y_{ij}{V}_{ij}^m-a}. \end{eqnarray*}

Lower ${R}_{ij}^{\ast }$ values indicate stronger competitive ability under the limitation of resource $j$ (Table 1).

The realized growth rate is then given by Liebig’s Law as


(6) \begin{eqnarray*} {\mu}_i=\underset{j}{\min}\left({\mu}_{ij}\right), \end{eqnarray*}

corresponding to the most limiting essential resource. Because the realized growth rate cannot exceed the rate supported by the limiting resource, we adjust the uptake of all essential nutrients accordingly. The realized uptake rate on resource $j$ is therefore


(7) \begin{eqnarray*} {V}_{ij}^r=\frac{\mu_i}{y_{ij}}. \end{eqnarray*}

Thus, ${V}_{ij}^m$ defines the potential maximum uptake, while the realized uptake ${V}_{ij}^r$ remains consistent with the growth rate imposed by the limiting resource, as required in a Liebig-type consumer–resource model.

Only external supply of OM and ${\mathrm{NO}}_3^{-}$ (${s}_1$ and ${s}_2$) is provided. Other metabolites are produced according to the excretion matrix ${E}_{ij}$, given by


(8) \begin{eqnarray*} {\displaystyle \begin{array}{@{}l@{}}\begin{array}{@{}ccccc@{}}\mathrm{O}\mathrm{M} & {\mathrm{N}\mathrm{O}}_3^{-} & {\mathrm{N}\mathrm{O}}_2^{-}& {\mathrm{N}}_2\mathrm{O}\ & {\mathrm{N}\mathrm{H}}_4^{+}\end{array}\\{}{E}_{ij}=\left(\begin{array}{@{}ccccc@{}}0& 0& {\mu}_1/{y}_{12}& 0& {e}_{15}\\{}0& 0& 0& {\mu}_2/{y}_{22}& {e}_{25}\\{}0& 0& 0& 0& {e}_{35}\\{}0& 0& 0& 0& {e}_{45}\\{}0& 0& 0& 0& 0\\{}0& 0& 0& {\mu}_6/{y}_{63}& {e}_{65}\\{}0& 0& 0& 0& {e}_{75}\end{array}\right)\begin{array}{@{}l@{}}{B}_1:{\mathrm{N}\mathrm{O}}_3^{-}\to{\mathrm{N}\mathrm{O}}_2^{-}\\{}{B}_2:{\mathrm{N}\mathrm{O}}_3^{-}\to{\mathrm{N}}_2\mathrm{O}\\{}{B}_3:{\mathrm{N}\mathrm{O}}_2^{-}\to{\mathrm{N}}_2\mathrm{O}\to{\mathrm{N}}_2\\{}{B}_4:{\mathrm{N}}_2\mathrm{O}\to{\mathrm{N}}_2\\{}{B}_5:{\mathrm{N}\mathrm{O}}_2^{-}+{\mathrm{N}\mathrm{H}}_4^{+}\to{\mathrm{N}}_2\\{}{B}_6:{\mathrm{N}\mathrm{O}}_2^{-}\to{\mathrm{N}}_2\mathrm{O}\\{}{B}_7:{\mathrm{N}\mathrm{O}}_3^{-}\to \! {\mathrm{N}\mathrm{O}}_2^{-}\to\! {\mathrm{N}}_2\mathrm{O}\to\! {\mathrm{N}}_2\end{array}\end{array}} \end{eqnarray*}

${\mathrm{NH}}_4^{+}$ is excreted as a byproduct of OM remineralization by heterotrophs, determined by the C:N of the OM and the electron-balanced budget for each metabolic functional type [20]. Assuming realistic ranges of bulk C:N of OM in the ocean, the supply rate of ${\mathrm{NH}}_4^{+}$, ${\sum}_{i=1}^7{e}_{i5}{B}_i$, is 70%–86% of the supply rate of OM (${s}_1$), and our model results are qualitatively consistent across this range.

We use the trait values from Sun *et al*. [20], which estimates the biomass yields from underlying redox chemistry and energetic constraints [23], and shows consistency with observations (Table 1).

### Simulation setup and analysis

To explore how nutrient regimes shape coexistence, we systematically vary the supply rates of OM and ${\mathrm{NO}}_3^{-}$ across ecologically relevant ranges (Supplementary Material, [20]). We then analyze the community composition at ecological equilibrium emerging from each of the supply rate combinations. For each of the resource supply rate ratios, we analyze the outcome of steady, rather than time-varying, supply rates to isolate the mechanistic effects of metabolic dependencies and resource competition. We recognize, however, that natural systems often exhibit substantial spatial and temporal variability in resource availability, and that such variability can promote coexistence beyond that predicted under constant conditions [8, 9, 24]. Additional mechanisms, such as density-dependent effect [25], may also promote diversity. In general, incorporating time-varying resource supply rates would expand the resulting ranges of coexistences. However, exploring these impacts is beyond the scope of the present study.

We use invasion analysis to determine coexistence conditions in a given resource environment. Specifically, graphical analyses of zero net growth isoclines (ZNGIs) and consumption vectors visualizes competitive outcomes and dependent interactions among groups [7]. The consumption vector characterizes the relative uptake of essential resources and delineates the regions of coexistence and dominance among groups [7, 8, 26]. In our framework, it is defined as the ratio of yields on two essential substrates. For example, for the ${\mathrm{NO}}_3^{-}\to{\mathrm{NO}}_2^{-}$ denitrifier that utilizes OM and ${\mathrm{NO}}_3^{-}$ as essential substrates, the consumption vector is given by (see derivation of Equation S20 in the Supplementary Material):


(9) \begin{eqnarray*} {c}_1=\frac{y_{1,\mathrm{OM}}}{y_{1,{\mathrm{NO}}_3^{-}}}. \end{eqnarray*}

When multiple populations coexist, the consumption vector reflects the demand of the community as a whole, rather than any one individual population. Below, we evaluate how the consumption vectors change to reflect community consumption when different recipients are introduced to examine the resulting shifts in coexistence patterns.

We also address broader biogeochemical outcomes by examining how microbial interactions determine the pathways of nitrogen loss at steady state. Nitrogen is considered lost when converted to gaseous products (${\mathrm{N}}_2$ or ${\mathrm{N}}_2\mathrm{O}$).

## Results and discussion

We analyze the resulting complex interactions by incrementally constructing the system from simpler subsystems. We start with two microbial types, the ${\mathrm{NO}}_3^{-}\to{\mathrm{NO}}_2^{-}$ denitrifier (${B}_1$) and the ${\mathrm{N}\mathrm{O}}_3^{-}\to{\mathrm{N}}_2\mathrm{O}$ denitrifier (${B}_2$), as all other types depend on production from one of these two types (except for the complete denitrifier (${B}_7$), discussed below). This stepwise approach disentangles the quantitative principles that govern microbial coexistence, exclusion, and resource partitioning. Furthermore, simplified microbial consortia are central to engineered ecosystems, such as wastewater treatment facilities, where specific functional groups—particularly denitrifiers and anammox bacteria—are selectively enriched to maximize nitrogen removal efficiency [23, 27], and so understanding the interactions of these simplified consortia could be useful for these contexts [28].

### Baseline system: two ${\mathbf{NO}}_{\mathbf{3}}^{-}$-reducing competitors

**Figure 2:**
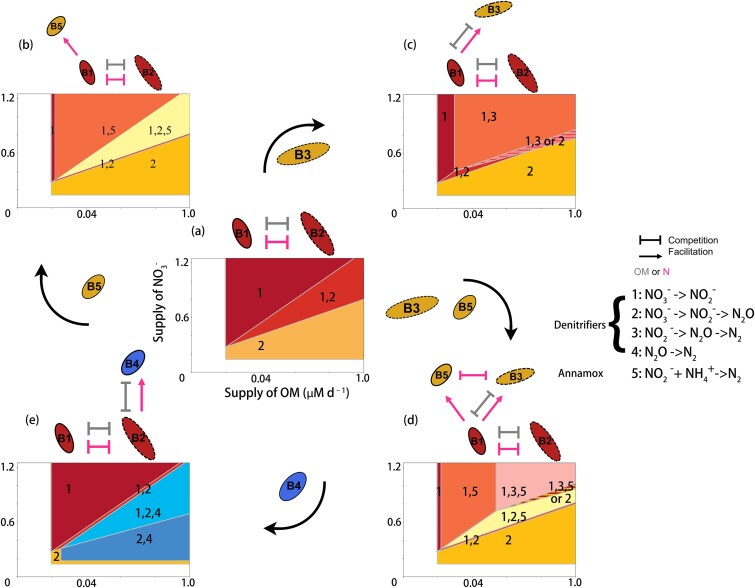
Competitive outcomes as a function of OM and ${\mathrm{NO}}_3^{-}$ supply rates across systems. Outcomes are shown in the OM–${\mathrm{NO}}_3^{-}$ supply space for the baseline system (a, center panel) and for alternative subsystems (b–e, surrounding panels).

We begin by analyzing what we term the baseline system of the two heterotrophs competing for OM and ${\mathrm{NO}}_3^{-}$: the ${\mathrm{NO}}_3^{-}\to{\mathrm{NO}}_2^{-}$ denitrifier (${B}_1$) reducing ${\mathrm{NO}}_3^{-}$ to ${\mathrm{NO}}_2^{-}$ and the ${\mathrm{N}\mathrm{O}}_3^{-}\to{\mathrm{N}}_2\mathrm{O}$ denitrifier (${B}_2$) reducing ${\mathrm{NO}}_3^{-}$ directly to ${\mathrm{N}}_2\mathrm{O}$ (Fig. 2a). Trait-based parameterization using the underlying energetics reveals a trade-off in their resource requirements [20]: the ${\mathrm{NO}}_3^{-}\to{\mathrm{NO}}_2^{-}$ reducer has a lower subsistence requirement and is a better competitor for OM (${R}_{1,\mathrm{OM}}^{\ast }<{R}_{2,\mathrm{OM}}^{\ast }$) but has higher requirement and is a weaker competitor for ${\mathrm{NO}}_3^{-}$ (${R}_{1,{\mathrm{NO}}_3^{-}}^{\ast }>{R}_{2,{\mathrm{NO}}_3^{-}}^{\ast }$) compared with the ${\mathrm{N}\mathrm{O}}_3^{-}\to{\mathrm{N}}_2\mathrm{O}$ reducer (Table 1). As a result, their consumption vectors (${c}_1=\frac{y_{1,\mathrm{OM}}}{y_{1,{\mathrm{NO}}_3^{-}}}$, ${c}_2=\frac{y_{2,\mathrm{OM}}}{y_{2,{\mathrm{NO}}_3^{-}}}$) form a coexistence region in the OM: ${\mathrm{NO}}_3^{-}$ supply space (Fig. 2a) [7, 8, 26]. When the OM: ${\mathrm{NO}}_3^{-}$ supply ratio is small, the ${\mathrm{NO}}_3^{-}\to{\mathrm{NO}}_2^{-}$ reducer dominates. When the OM: ${\mathrm{NO}}_3^{-}$ supply ratio is large, the ${\mathrm{N}\mathrm{O}}_3^{-}\to{\mathrm{N}}_2\mathrm{O}$ reducer prevails. At intermediate supply ratios, both groups coexist (Fig. 2a).

**Figure 3:**
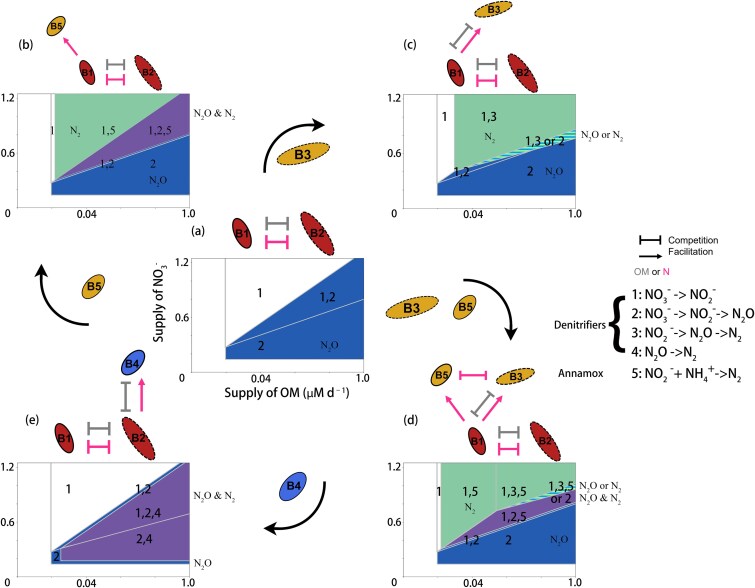
Nitrogen loss pathways as a function of OM and ${\mathrm{NO}}_3^{-}$ supply rates. Pathways are mapped in the OM–${\mathrm{NO}}_3^{-}$ supply space for the baseline system (a, center panel) and for the corresponding subsystems (b–e, surrounding panels).

In this subsystem, because only the ${\mathrm{N}\mathrm{O}}_3^{-}\to{\mathrm{N}}_2\mathrm{O}$ reducer directly produces ${\mathrm{N}}_2\mathrm{O}$, nitrogen loss occurrs only when OM supply is sufficiently high to sustain the ${\mathrm{N}\mathrm{O}}_3^{-}\to{\mathrm{N}}_2\mathrm{O}$ reducer’s persistence (Fig. 3a).

**Figure 4:**
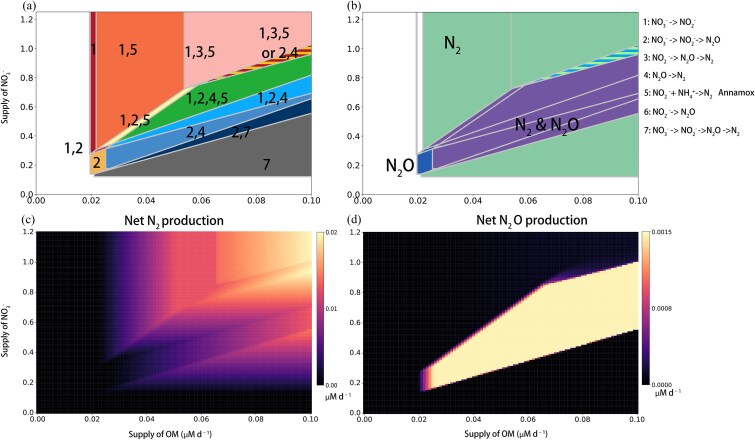
Graphical analysis of functional group coexistence (a), nitrogen loss pathways (b), and the net microbial ${\mathrm{N}}_2$ and ${\mathrm{N}}_2\mathrm{O}$ production (i.e. microbial sources minus microbial sinks) (c and d). In panel (b), regions showing both ${\mathrm{N}}_2$ and ${\mathrm{N}}_2\mathrm{O}$ correspond to steady states in which ${\mathrm{N}}_2\mathrm{O}$ production is balanced by its consumption; in natural environments (e.g. the ocean), such conditions nonetheless create the potential for ${\mathrm{N}}_2\mathrm{O}$ emission.

A third denitrifier—the complete denitrifier, ${B}_7$—is a direct competitor with ${B}_1$ and ${B}_2$, using both OM and ${\mathrm{NO}}_3^{-}$. However, again reflecting the underlying energetics, it has a competitive advantage in utilizing ${\mathrm{NO}}_3^{-}$ (a lower ${R}_{7,{\mathrm{NO}}_3^{-}}^{\ast }$ than those of ${B}_1$ and ${B}_2$), allowing it to outcompete ${B}_1$ and ${B}_2$ under more ${\mathrm{NO}}_3^{-}$-limited conditions [20]. Consequently, ${B}_7$ dominates the community when ${\mathrm{NO}}_3^{-}$ availability is low (Fig. 4a). Because its coexistence region lies largely outside the primary parameter space of the other groups, we include it in the full system for completeness but do not analyze it in detail.

### Recipient groups restructure competitive outcomes

We next examine how introducing recipient functional groups alters these baseline dynamics. Recipients require substrates produced by feeders, creating dependent links, but often also compete with feeders or feeders’ competitors for OM. These dual roles restructure coexistence outcomes in qualitatively different ways depending on resource overlap.

#### Adding anammox: a neutral recipient

We next add anammox bacteria to the baseline system (Fig. 2b). Anammox can be theoretically limited by either ${\mathrm{NO}}_2^{-}$, produced by the ${\mathrm{NO}}_3^{-}\to{\mathrm{NO}}_2^{-}$ denitrifier, or ${\mathrm{NH}}_4^{+}$ derived from OM remineralization (Equation (4)). In either case, anammox does not impact the direct competition between the two ${\mathrm{NO}}_3^{-}$ reducers and thus does not alter the competitive boundaries between them.

However, anammox, as a recipient, is directly limited by the activity of the denitrifiers as feeders. In this subsystem, ${\mathrm{NH}}_4^{+}$ supply is sufficient for anammox to persist across the domain (${s}_{{\mathrm{NH}}_4^{+}}>{R}_{5,{\mathrm{NH}}_4^{+}}^{\ast }$). So, the existence of anammox only relies on the production of ${\mathrm{NO}}_2^{-}$ by the ${\mathrm{NO}}_3^{-}\to{\mathrm{NO}}_2^{-}$ denitrifier (${B}_1$). Once ${B}_1$ reaches sufficient biomass, anammox can persist. Thus, there is a minimum requirement for the density of ${B}_1$ to produce enough ${\mathrm{NO}}_2^{-}$ to sustain anammox, as follows.

A general criteria for the minimum equilibrium density of a feeder type required to sustain a recipient type is given by (Supplementary Material, Equation S15):


(10) \begin{eqnarray*} {\hat{B}}_{\mathrm{Feeder}}>\frac{a{y}_{\mathrm{Feeder},\mathrm{Converted}\ \mathrm{resource}}}{\mu_{\mathrm{Recipient}}} \end{eqnarray*}

Thus, the equilibrium density of ${B}_1$ required for anammox to invade is ${\hat{B}}_1>\frac{a{y}_{12}}{\mu_5}$ where ${y}_{12}$ is the yield of the feeder ${B}_1$ using ${\mathrm{NO}}_3^{-}$ (${R}_2$) and ${\mu}_5$ is the realized growth rate of anammox (${B}_5$) when limited by ${\mathrm{NO}}_2^{-}$ (${R}_3$). Equation (10) shows that at a steady state, the requirement of the biomass of the feeder increases with the yield of the feeder on ${\mathrm{NO}}_3^{-}$, as the ratio of biomass synthesis to ${\mathrm{NO}}_3^{-}$ reduction increases, and decreases with the growth rate of anammox. This balance is reflected in the graphic analysis for OM and ${\mathrm{NO}}_3^{-}$, even though anammox do not directly require either substrate. We can use Equation (10) to derive the ZNGI of ${B}_5$ for OM and ${\mathrm{NO}}_3^{-}$, which is above the ZNGI of ${B}_1$ (Fig. 2b and Fig. S1), showing that anammox can only persist when the ${\mathrm{NO}}_3^{-}\to{\mathrm{NO}}_2^{-}$ denitrifier (${B}_1$) establishes a sufficient biomass.

Anammox expands the region of nitrogen loss by introducing an ${\mathrm{N}}_2$ production pathway to the previous system (Fig. 3b). This case demonstrates that recipients can broaden the functional consequences of microbial communities without altering coexistence boundaries among their feeders and the feeders’ competitor.

#### Adding ${\mathbf{N}\mathbf{O}}_{\mathbf{2}}^{-}\mathbf{\to}{\mathbf{N}}_{\mathbf{2}}$ denitrifiers: priority effects via intensified competition

We next introduce the ${\mathrm{N}\mathrm{O}}_2^{-}\to{\mathrm{N}}_2$ denitrifier (${B}_3$) into the baseline system, which produces a distinct outcome (Fig. 2c). Like anammox, ${B}_3$ depends on ${\mathrm{NO}}_2^{-}$ supplied by ${B}_1$ and requires a minimum threshold density of ${B}_1$, given by ${\hat{B}}_1\ge \frac{ay_{12}}{\mu_3}$ (Equation (10)). Unlike anammox, however, ${B}_3$ also consumes OM, and, reflecting the underlying energetics, has the lowest subsistence requirement for OM among the three groups (${R}_{3,\mathrm{OM}}^{\ast }<{R}_{1,\mathrm{OM}}^{\ast }<{R}_{2,\mathrm{OM}}^{\ast }$) (Table 1). To coexist with ${B}_1$, ${B}_3$must remain limited by ${\mathrm{NO}}_2^{-}$; otherwise, if it were limited by OM, it would exclude the ${\mathrm{NO}}_2^{-}$ reducer (which would only happen if ${\mathrm{NO}}_2^{-}$ is sufficiently supplied from a different source).

Once established, the feeder–recipient pair (${B}_1$ and ${B}_3$) forms a tightly coupled consortium that depletes more OM relative to ${\mathrm{NO}}_3^{-}$ than the ${\mathrm{NO}}_3^{-}\to{\mathrm{NO}}_2^{-}$ reducer alone. This is reflected in the consortium’s consumption vector ${c}_{13}$ (see Equation S19 for derivation):


(11) \begin{eqnarray*} {c}_{13}=\frac{c_1}{\left(1+{c}_1/{c}_3\right)} \end{eqnarray*}

which is a function of the two individual consumption vectors, ${c}_1=\frac{y_{1,\mathrm{OM}}}{y_{1,{\mathrm{NO}}_3^{-}}}$ and ${c}_3=\frac{y_{3,\mathrm{OM}}}{y_{3,{\mathrm{NO}}_2^{-}}}$. Equation (11) shows that ${c}_{13}$ is smaller than ${c}_1$, indicating a greater relative consumption of OM compared to the ${\mathrm{N}\mathrm{O}}_3^{-}\to{\mathrm{N}}_2\mathrm{O}$ denitrifier. Consequently, the consortium exerts a stronger suppressive effect on the ${\mathrm{N}\mathrm{O}}_3^{-}\to{\mathrm{N}}_2\mathrm{O}$ denitrifier under OM-limited conditions, thereby narrowing their niche (Fig. 2a and c).

Furthermore, the consortium’s stronger resource consumption on OM can generate priority effects in cases where ${\mathrm{N}\mathrm{O}}_3^{-}\to{\mathrm{N}}_2\mathrm{O}$ and ${\mathrm{NO}}_3^{-}\to{\mathrm{NO}}_2^{-}$ coexist in the baseline system, which means that the outcome depends on the order of establishment (Fig. 2c and Fig. S1, Equations S19and  S20) [7, 26, 29]. If the feeder–recipient pair becomes established first, it prevents invasion by the ${\mathrm{N}\mathrm{O}}_3^{-}\to{\mathrm{N}}_2\mathrm{O}$ reducer. If the ${\mathrm{N}\mathrm{O}}_3^{-}\to{\mathrm{N}}_2\mathrm{O}$ reducer arrives first, it can exclude the pair. Therefore, the niche space of the ${\mathrm{N}\mathrm{O}}_3^{-}\to{\mathrm{N}}_2\mathrm{O}$ denitrifier is potentially narrowed (Fig. 2 ac).

Biogeochemically, the ${\mathrm{N}\mathrm{O}}_2^{-}\to{\mathrm{N}}_2$ recipient expands the region of nitrogen loss by enabling ${\mathrm{N}}_2$ production under OM-sufficient conditions, similar to the subsystem containing anammox (Fig. 3c). In the priority-effect region, the dominant form of nitrogen loss depends on the community’s assembly history, yielding either ${\mathrm{N}}_2$ from the feeder–dependent pair or ${\mathrm{N}}_2\mathrm{O}$ from the ${\mathrm{N}\mathrm{O}}_3^{-}\to{\mathrm{N}}_2\mathrm{O}$ denitrifier.

#### Interactions among the two recipients of the ${\mathbf{NO}}_{\mathbf{3}}^{-}\mathbf{\to}{\mathbf{NO}}_{\mathbf{2}}^{-}$ reducer

We next add both recipients of the ${\mathrm{NO}}_3^{-}\to{\mathrm{NO}}_2^{-}$ reducer—anammox and the ${\mathrm{N}\mathrm{O}}_2^{-}\to{\mathrm{N}}_2$ denitrifier—to the baseline system (Fig. 2d). As above, the supply of ${\mathrm{NH}}_4^{+}$ from OM remineralization is sufficient for anammox, so anammox still depends on the ${\mathrm{NO}}_3^{-}\to{\mathrm{NO}}_2^{-}$ denitrifier (Equation (10)) for ${\mathrm{NO}}_2^{-}$, but now competes for it against the ${\mathrm{N}\mathrm{O}}_2^{-}\to{\mathrm{N}}_2$ denitrifier.

Critically, we here assume that anammox is a superior competitor for ${\mathrm{NO}}_2^{-}$ compared with the heterotrophic ${\mathrm{N}\mathrm{O}}_2^{-}\to{\mathrm{N}}_2$ denitrifier, following our best estimate given observed affinities (Table 1; [20]). Thus, anammox has a lower minimum subsistence concentration (${R}_{5,\mathrm{N}{\mathrm{O}}_2^{-}}^{\ast }<{R}_{3,\mathrm{N}{\mathrm{O}}_2^{-}}^{\ast }$), and so, under ${\mathrm{NO}}_2^{-}$ limitation, anammox can exclude the competing denitrifier. With this assumption, in Fig. 2d, anammox first becomes sustainable as the supply of ${\mathrm{NO}}_3^{-}$ increases relative to OM, before the competing denitrifier (the red-orange and yellow regions in Fig. 2d). Then, once the growth of anammox becomes limited by ${\mathrm{NH}}_4^{+}$ rather than ${\mathrm{NO}}_2^{-}$, the denitrifier can invade and coexist with anammox and the feeder (pink region in Fig. 2d). Thus, anammox and the ${\mathrm{N}\mathrm{O}}_2^{-}\to{\mathrm{N}}_2$ denitrifier coexist as long as the ${\mathrm{NO}}_2^{-}$ production is sufficient for the ${\mathrm{N}\mathrm{O}}_2^{-}\to{\mathrm{N}}_2$ denitrifier’s persistence at high supply of OM and ${\mathrm{NO}}_3^{-}$ (Fig. S8).

Alternatively, if anammox were an inferior competitor for ${\mathrm{NO}}_2^{-}$ than the ${\mathrm{N}\mathrm{O}}_2^{-}\to{\mathrm{N}}_2$ denitrifier [30], it would be competitively excluded throughout the domain (Fig. S9), preventing any possibility of coexistence. Indeed, Fadum *et al*. [30] considered both scenarios (with anammox as a superior and an inferior competitor for ${\mathrm{NO}}_2^{-}$) because, despite evidence suggesting anammox having a higher affinity for ${\mathrm{NO}}_2^{-}$ (Table 1; [20]), it remains unclear which scenario is more realistic, particularly because the controls on ${\mathrm{NO}}_2^{-}$ concentrations in anoxic zones remain unresolved.

This subsystem also results in a distinct N loss pattern. Unlike the ${\mathrm{N}\mathrm{O}}_2^{-}\to{\mathrm{N}}_2$ denitrifier, which assists its feeder suppress the niche of the ${\mathrm{N}\mathrm{O}}_3^{-}\to{\mathrm{N}}_2\mathrm{O}$ denitrifier, anammox exerts no such effect. By suppressing the ${\mathrm{N}\mathrm{O}}_2^{-}\to{\mathrm{N}}_2$ denitrifier, anammox indirectly benefits the feeder’s competitor (${\mathrm{N}\mathrm{O}}_3^{-}\to{\mathrm{N}}_2\mathrm{O}$ denitrifier), expanding its niche. Consequently, the coexistence space of ${\mathrm{NO}}_3^{-}\to{\mathrm{NO}}_2^{-}$ and ${\mathrm{N}\mathrm{O}}_3^{-}\to{\mathrm{N}}_2\mathrm{O}$ reducers broadens (Fig. 2a, d). This, in turn, leads to an expanded N-loss region characterized by mixed ${\mathrm{N}}_2$ and ${\mathrm{N}}_2\mathrm{O}$ production (Fig. 3d).

#### Adding ${\mathbf{N}}_{\mathbf{2}}\mathbf{O}\mathbf{\to }{\mathbf{N}}_{\mathbf{2}}$ denitrifiers: new recipients erode feeder dominance

For the final subset of the full system, we add the ${\mathrm{N}}_2\mathrm{O}\to{\mathrm{N}}_2$ denitrifier (${B}_4$) into the baseline system (Fig. 2e). The ${\mathrm{N}}_2\mathrm{O}\to{\mathrm{N}}_2$ denitrifier depends on ${\mathrm{N}}_2\mathrm{O}$ produced by the ${\mathrm{N}\mathrm{O}}_3^{-}\to{\mathrm{N}}_2\mathrm{O}$ reducer and consumes OM more efficiently than their feeder (${R}_{4,\mathrm{OM}}^{\ast }<{R}_{2,\mathrm{OM}}^{\ast }$) [20]. This subsystem differs fundamentally from the previous one because the feeder, the ${\mathrm{N}\mathrm{O}}_3^{-}\to{\mathrm{N}}_2$O denitrifier (${B}_2$), is OM-limited and is therefore a weaker competitor for OM when coexisting with the ${\mathrm{NO}}_3^{-}\to{\mathrm{NO}}_2^{-}$ denitrifier (${B}_1$). Although both ${B}_3$ and ${B}_4$ are superior OM competitors relative to ${B}_1$ and ${B}_2$, respectively, ${B}_1$ is not OM-limited in this system, whereas ${B}_2$ is. Therefore, the emergent consortia behave qualitatively differently across the two subsystems. In the ${B}_1$–${B}_2$–${B}_3$ system, ${B}_3$ expands ${B}_1$’s realized niche by eroding ${B}_2$’s niche through intensified OM competition. In contrast, when the ${N}_2$O$\to{N}_2$ denitrifier (${B}_4$) is introduced into the ${B}_1$–${B}_2$ baseline, ${B}_4$—despite being metabolically downstream and dependent on ${B}_2$—further increases total OM demand, thereby intensifying OM limitation for ${B}_2$ and indirectly facilitating the expansion of ${B}_1$’s niche. This result is reflected by the consortium-level consumption vector, given by (see Equation S94 for details) ${c}_{24}=\frac{c_2}{\left(1+{c}_2/{c}_4\right)}$, being smaller than ${c}_2$, indicating a higher relative OM demand. This shift effectively reduces the competitive advantage of the feeder and expands the niche space of the ${\mathrm{NO}}_3^{-}\to{\mathrm{NO}}_2^{-}$ denitrifier (Fig. 2e).

Biogeochemically, the ${\mathrm{N}}_2\mathrm{O}\to{\mathrm{N}}_2$ denitrifier converts ${\mathrm{N}}_2\mathrm{O}$ to ${\mathrm{N}}_2$. In the virtual chemostat, ${\mathrm{N}}_2\mathrm{O}$ acts as an intermediate, i.e. consumed by the ${\mathrm{N}}_2\mathrm{O}\to{\mathrm{N}}_2$ denitrifier as quickly as it is produced. Thus, nitrogen loss occurs primarily as ${\mathrm{N}}_2$ while still maintaining the potential for ${\mathrm{N}}_2\mathrm{O}$ escape (Fig. 3e).

### The full ecological system

We next analyze the “full system” with all seven functional types introduced (Fig. 4a). We describe the overall ecological dynamics giving rise to these communities followed by their nitrogen loss patterns.

#### Ecological dynamics

The system does not support the coexistence of all seven functional types at any point (Fig. 4a). Rather, multiple distinct communities emerge, structured by trade-offs, priority effects, and indirect facilitation. Generally, the changes in community composition follow the broad patterns outlined in Sun *et al*. [20]: as OM:${\mathrm{NO}}_3^{-}$ supply increases, and the system shifts from OM limitation to ${\mathrm{NO}}_3^{-}$ limitation, pathway length increases for the ${\mathrm{NO}}_3^{-}$-consuming denitrifiers, in accordance with the underlying energetics (${R}_{1,\mathrm{OM}}^{\ast }<{R}_{2,\mathrm{OM}}^{\ast }<{R}_{7,\mathrm{OM}}^{\ast }$, Table 1). Thus, the complete denitrifier (${B}_7$) only persists with sufficient supply of OM, coexisting with the ${\mathrm{N}\mathrm{O}}_3^{-}\to{\mathrm{N}}_2\mathrm{O}$ denitrifier (${B}_2$) over a small domain (dark blue triangle in Fig. 4), and is consistently outcompeted by more specialized groups when the supply of ${\mathrm{NO}}_3^{-}$ increases. The ${\mathrm{NO}}_3^{-}\to{\mathrm{NO}}_2^{-}$ and ${\mathrm{N}\mathrm{O}}_2^{-}\to{\mathrm{N}}_2$ denitrifier consortia exists over much of the domain, in consistency with Sun *et al*. [20] and characteristic of much of the OM-limited open ocean. Because ${\mathrm{NO}}_2^{-}$ remains limiting throughout the domain, the ${\mathrm{N}\mathrm{O}}_2^{-}\to{\mathrm{N}}_2$ denitrifier excludes the other recipient–feeder pair (${\mathrm{N}\mathrm{O}}_3^{-}\to{\mathrm{N}}_2\mathrm{O}\ \mathrm{and}\ {\mathrm{N}}_2\mathrm{O}\to{\mathrm{N}}_2\ \mathrm{denitrifiers}$). Also as in Sun *et al*. [20], the ${\mathrm{N}\mathrm{O}}_2^{-}\to{\mathrm{N}}_2\mathrm{O}$ denitrifier (${B}_6$) is competitively excluded throughout the domain because it is the weakest competitor for ${\mathrm{NO}}_2^{-}$, suggesting that persistence of this denitrifier module requires alleviation from ${\mathrm{NO}}_2^{-}$-limited growth.

However, the 2D of our analysis results in new regimes and nuances not addressed in Sun *et al*. [20]. First, in a narrow region of resource ratios, priority effects between functional consortiums matter (the red and orange striped region in Fig. 4a), meaning that the ecological outcome depends on the history of the community assembly. In a biogeochemical model that resolves N-cycling by dynamic functional types in this way, this implies that model solutions may be sensitive to initial biomass conditions, depending on the extent to which the system occupies this region of resource–ratio space.

Second, our analysis identifies the indirect impacts of anammox on the denitrifier community, as well as its broader niche. As above, ${\mathrm{NH}}_4^{+}$ supply is sufficient to support anammox across the domain, and so anammox relies on ${\mathrm{NO}}_2^{-}$ production from the ${\mathrm{NO}}_3^{-}\to{\mathrm{NO}}_2^{-}$ denitrifier. The ${\mathrm{NO}}_3^{-}\to{\mathrm{NO}}_2^{-}$ denitrifier exhibits a wider niche across the 2D space because in only a subset of the space does it reach the minimum biomass needed to sustain anammox (Equation (10)). As this minimum biomass is attained, anammox growth is sustained but ${\mathrm{NO}}_2^{-}$-limited, outcompeting the ${\mathrm{N}\mathrm{O}}_2^{-}\to{\mathrm{N}}_2$ denitrifier. Then, as ${\mathrm{NO}}_2^{-}$ supply increases, further growth of anammox is instead limited by ${\mathrm{NH}}_4^{+}$, which allows the ${\mathrm{N}\mathrm{O}}_2^{-}\to{\mathrm{N}}_2$ denitrifier to coexist (as in Section Recipient groups restructure competitive outcomes). This results in a broader metabolic niche for anammox relative to the ${\mathrm{N}\mathrm{O}}_2^{-}\to{\mathrm{N}}_2$ denitrifier, discussed in more detail below.

However, in the full system, another dynamic is at play: while the ${\mathrm{NO}}_3^{-}\to{\mathrm{NO}}_2^{-}$ and ${\mathrm{N}\mathrm{O}}_2^{-}\to{\mathrm{N}}_2$ denitrifier consortium excludes the other recipient–feeder pair $({\mathrm{N}\mathrm{O}}_3^{-}\to{\mathrm{N}}_2\mathrm{O}$and${\mathrm{N}}_2\mathrm{O}\to{\mathrm{N}}_2$ denitrifiers) in the OM-limited regime, anammox bacteria mitigate this dominance by outcompeting the ${\mathrm{N}\mathrm{O}}_2^{-}\to{\mathrm{N}}_2$ denitrifier under ${\mathrm{NO}}_2^{-}$ limitation (${R}_{5,{\mathrm{NO}}_2^{-}}^{\ast }<{R}_{3,{\mathrm{NO}}_2^{-}}^{\ast }$), benefiting the recipient–feeder pair (${\mathrm{N}\mathrm{O}}_3^{-}\to{\mathrm{N}}_2\mathrm{O}$ and ${\mathrm{N}}_2\mathrm{O}\to{\mathrm{N}}_2$ denitrifiers) and facilitating broader coexistence among denitrifiers. In other words, anammox extends the regime in which ${\mathrm{N}}_2\mathrm{O}$ is cycled as an intermediate. The extent of the benefit depends on the severity of ammonium limitation in anammox bacteria.

#### A broad niche for anammox

A striking outcome of our model is the disparity in niche breadth between anammox and ${\mathrm{NO}}_2^{-}$-reducing denitrifiers. Anammox occupies a wider niche in Fig. 4a than the ${\mathrm{NO}}_2^{-}$-consuming denitrifiers. This asymmetry stems from our evidence-based assumption that anammox possesses a lower ${\mathrm{NO}}_2^{-}$ requirement (${R}_{{\mathrm{NO}}_2^{-}}^{\ast }$) than its denitrifying competitors (Table 1; [20], but see above mention of the consideration of both scenarios by Fadum *et al*. [30]). Consequently, if this competitive advantage holds, and if ${\mathrm{NO}}_2^{-}$ is indeed limiting in some anoxic areas, such as around the periphery of an anoxic zone, anammox can, in principle, maintain a broader nutrient niche. This constitutes one hypothesis for why anammox is more frequently detected in OMZs, even in regions where denitrification-driven ${\mathrm{N}}_2$ production is prevalent [31–34]. While denitrifiers are biogeochemically vital, their narrower niches likely render them less consistently detectable across varying environmental gradients. This hypothesis could be tested by specifically examining competition for ${\mathrm{NO}}_2^{-}$ between anammox and ${\mathrm{NO}}_2^{-}$-consuming denitrifiers.

#### Patterns of nitrogen loss

We next analyze the nitrogen loss patterns of the full system. Sustaining any denitrifier population requires minimal supplies of both OM and ${\mathrm{NO}}_3^{-}$. Below these thresholds, no denitrifiers considered can persist in our model and nitrogen is conserved. Otherwise, the OM: ${\mathrm{NO}}_3^{-}$ supply ratio dictates the magnitude and form of nitrogen loss (Fig. 3b). Similar to Sun *et al*. [20], the cycling of ${\mathrm{N}}_2\mathrm{O}$ as an intermediate along the denitrification pathway is sustained at the intermediate OM: ${\mathrm{NO}}_3^{-}$ ratios (purple area in Fig. 4b), reflecting the above patterns in pathway length as a function of resource supply ratios. However, our results differ from the previous as follows.

#### N_2_O production but not consumption under co-limitation

In general, increasing OM and ${\mathrm{NO}}_3^{-}$ supply enhances total nitrogen loss via ${\mathrm{N}}_2$ production (Fig. 4c). However, we identify an area in parameter space where ${\mathrm{N}}_2$ production is reduced due to diversion of flux toward ${\mathrm{N}}_2\mathrm{O}$ through incomplete denitrification by ${\mathrm{N}\mathrm{O}}_3^{-}\to{\mathrm{N}}_2\mathrm{O}$ denitrifiers, consistent with the corresponding persistence region (Fig. 4a). Accordingly, ${\mathrm{N}}_2\mathrm{O}$ flux is confined to this area where these microbes are maintained (Fig. 4d). Within the central “diamond” region (the blue area in Fig. 4b), ${\mathrm{N}}_2\mathrm{O}$ consumption is not sustained. Thus, its concentration is governed by the balance between microbial production and physical transport rather than biological uptake. Consequently, in natural systems represented by the diamond region, ${\mathrm{N}}_2\mathrm{O}$ may accumulate or be transported by the ocean circulation. Once ${\mathrm{N}}_2\mathrm{O}$ consumption becomes viable, the steady-state ${\mathrm{N}}_2\mathrm{O}$ concentration is instead regulated by the ${R}^{\ast }$ of the ${\mathrm{N}}_2\mathrm{O}$-reducing population, and so does not have the potential for accumulation.

This accumulation regime emerges as the low-supply limit of the broader ${\mathrm{N}}_2\mathrm{O}$-producing region described above. When both OM and ${\mathrm{NO}}_3^{-}$ supplies are low, denitrifiers persist at densities too low to support downstream reducers, so ${\mathrm{N}}_2\mathrm{O}$ is produced but not consumed (Fig. 4b, lower-left corner). While increased OM promotes both ${\mathrm{N}}_2\mathrm{O}$ production and consumption [35, 36], our results show this relationship is also contingent upon ${\mathrm{NO}}_3^{-}$ availability. When electron donors and acceptors are both limiting, production and consumption become decoupled, and the denitrification pathway proceeds only partially. As a result, ${\mathrm{N}}_2\mathrm{O}$ accumulates because the system lacks sufficient substrate flux to sustain complete reduction to ${\mathrm{N}}_2$, shifting both the magnitude and direction of net nitrogen flux.

## Synthesis and general principles

Our results reveal general principles for how recipient populations reshape microbial coexistence and ecosystem function. Depending on their competitive ability relative to feeders and competitors, recipients can reinforce, erode, or leave unaffected the ecological niches of their feeders.

Reinforcement of feeder dominance occurs when recipients specialize in resources that are not limiting to their feeders but are limiting to the feeder’s competitor. In these cases, the feeder–recipient association intensifies competitive exclusion of the competitor (e.g. the group of ${B}_1$, ${B}_2$, and ${B}_3$). In contrast, erosion of feeder dominance, by expanding feeder’s competitor niche, arises when recipients overlap strongly in resource use with their feeders but are more competitive for the resource that limits their feeders. This reduces the feeder’s competitive advantage and expands the niche of its competitor (e.g. the group of ${B}_1$, ${B}_2$, and ${B}_4$). Finally, intuitively, no change in the dominance of the feeder occurs when recipients rely on resources unrelated to their feeder’s limitation, leaving competitive boundaries unaffected (e.g. the group of ${B}_1$, ${B}_2$, and anammox ${B}_5$).

Together, these principles extend coexistence theory to multi-resource systems with dependencies. Our results are relevant for microbial interaction networks beyond just that of the marine nitrogen cycle, providing a framework for untangling the complexities. We have demonstrated that facilitation interacts with competition to shape both community assembly and biogeochemical function.

## Conclusion

We show that recipient populations fundamentally restructure competitive landscapes by modifying how limiting resources are partitioned. Rather than passive endpoints of a metabolic flux, recipients alter the constraints experienced by their feeders and other competing populations, reshaping coexistence boundaries. Whether these effects favor the persistence of feeders or their competitors depends on how recipient traits align with system-level resource limitation. Thus, ecological outcomes emerge from the joint structure of metabolic dependencies and resource competition rather than from pairwise interactions alone.

Beyond community structure, interaction-mediated shifts in coexistence propagate to ecosystem-scale nitrogen loss. By jointly varying OM and ${\mathrm{NO}}_3^{-}$ supply, we uncover emergent biogeochemical regimes that are not predictable from single-resource gradients or fixed supply ratios alone. These include a co-limitation regime favoring ${\mathrm{N}}_2$O production and regions where historical contingencies and priority effects shape pathway dominance. These results link microbial interaction structure directly to variability in nitrogen loss pathways.

More broadly, our framework highlights how microbial traits, metabolic dependencies, and multi-resource supply interact to regulate ecosystem-scale transformation of energy and nutrients. Because feeder–recipient interactions are ubiquitous, the principles identified here extend beyond aquatic anoxic zone N-cycling to diverse environments where microbial interactions and coexistence determine the fate of many elemental cycles, including soil redox gradients and host-associated microbiomes, where metabolic byproducts of one population serve as essential substrates for others, forming networks of directional metabolic dependencies [14, 37]. This perspective connects trait-based ecology organizing complex microbial communities [38, 39] with large-scale nutrient cycling.

## Supplementary Material

SupplementaryMaterials_v16_ycag134

## Data Availability

All data generated and analyzed during the current study were produced from simulation models. The simulated datasets and scripts used to generate and analyze them are available in the GitHub repository: https://github.com/xl0418/Dependency-Competition_Tradeoffs_and_N_Cycling_Project.
