# Supplementary material for: Dependency–competition tradeoffs structure microbial niches and nitrogen cycling
Source: ISME Commun. 2026 May 15;6(1):ycag134. doi: 10.1093/ismeco/ycag134 (PMC13235746; doi:10.1093/ismeco/ycag134)
Supplement: SupplementaryMaterials_v16_ycag134 [file supplementarymaterials_v16_ycag134.pdf]

Supplementary Material: Facilitation–Competition Trade-offs Structure Microbial Niches and Nitrogen Cycling

Liang Xu

This supplementary material provides the detailed theoretical framework and mathematical derivations supporting the findings presented in the main text. Specifically, we offer a comprehensive analysis of both the individual functional subsystems and the integrated consortia model. Included herein are step-by-step derivations of the governing equations, proofs of the steady-state solutions, and the analytical grounding for the conclusions reached in our study.

1 The Model of Two Feeder Species and One Recipient Species

We first consider a model of two species (species 1 and 2) competing for two resources (resource 1 and 2) and one recipient species (species 3) consuming a resource (resource 3) produced by species 1. The resource consumer model is given by

$$\frac{1}{B_1}\frac{dB_1}{dt} = \min\left(\mu_{11}R_1, \mu_{12}R_2\right) - m \tag{1}$$

$$\frac{1}{B_2}\frac{dB_2}{dt} = \min\left(\mu_{21}R_1, \mu_{22}R_2\right) - m \tag{2}$$

$$\frac{1}{B_3}\frac{dB_3}{dt} = \min\left(\mu_{31}R_1, \mu_{33}R_3\right) - m \tag{3}$$

$$\frac{dR_1}{dt} = a(s_1 - R_1) - V_{11}B_1 - V_{21}B_2 - V_{31}B_3 \tag{4}$$

$$\frac{dR_2}{dt} = a(s_2 - R_2) - V_{12}B_1 - V_{22}B_2 \tag{5}$$

$$\frac{dR_3}{dt} = (s_{13}(B_1) - aR_3) - V_{33}B_3 \tag{6}$$

where $V_{ij} = \frac{\min(\mu_{i1}R_1, \mu_{i2}R_2)}{y_{ij}}$ for species 1 and 2 and $V_{3j} = \frac{\min(\mu_{31}R_1, \mu_{33}R_3)}{y_{3j}}$. In a chemostat model, $m = a$ in the main text. For mathematical tractability, we use linear growth functional forms (LM) to approach Michaelis-Menten (MM) kinetics throughout the theoretical framework:

$$\mu_{LM} \approx \frac{\mu_{MM}}{K}. \tag{7}$$

This approximation is often used when the resource is at low density ($R < K$),

$$\mu_{LM}R \approx \mu_{MM}\frac{R}{R+K}. \tag{8}$$

There are several assumptions:

1. The recipient species (species 3) has to be limited by R_3 and has a lower $R^*_{31} < R^*_{11}$ on resource 1. Otherwise, species 3 will be excluded by its feeder B_1. Thus, at equilibrium $\min\left(\mu_{31}R_1, \mu_{33}R_3\right) = \mu_{33}\hat{R}_3$ and $V_{33} = \frac{\mu_{33}\hat{R}_3}{y_{33}}$.

2. B_1, B_2 have distinct advantage on utilizing resource 1 and 2. Assume that $R^*_{11} < R^*_{21}$ and $R^*_{22} < R^*_{12}$. So, species 1 is limited by resource 2 while species 2 is limited by resource 1, yielding $\min(\mu_{11}R_1, \mu_{12}R_2) = \mu_{12}\hat{R}_2$ and $\min(\mu_{21}R_1, \mu_{22}R_2) = \mu_{21}\hat{R}_1$. Similarly, $V_{1j} = \frac{\mu_{12}\hat{R}_2}{y_{1j}}$ and $V_{2j} = \frac{\mu_{21}\hat{R}_1}{y_{2j}}$.

3. Resource 3 is only produced by B_1. The supply is then a function of the density of B_1. One specific functional form is given by $\delta V_{12}B_1$ where a portion δ of R_2 up-taken by B_1 is converted to R_3. In the main text, we choose $\delta = 1$.

4. Therefore, at the steady state, the three species are limited by different resources, i.e., species 1 is limited by resource 2, species 2 is limited by resource 1 and species 3 is limited by resource 3. Thus, we have

$$\min(\mu_{11}R_1, \mu_{12}R_2) := \mu_1 = \mu_{12}\hat{R}_2 \tag{9}$$

$$\min(\mu_{21}R_1, \mu_{22}R_2) := \mu_2 = \mu_{21}\hat{R}_1 \tag{10}$$

$$\min(\mu_{31}R_1, \mu_{33}R_3) := \mu_3 = \mu_{33}\hat{R}_3 \tag{11}$$

$$\hat{R}_1 = \frac{m}{\mu_{21}}, \hat{R}_2 = \frac{m}{\mu_{12}}, \hat{R}_3 = \frac{m}{\mu_{33}} \tag{12}$$

At the steady-state, we can first calculate the relationship between B_1 and B_3 by assuming R_3 is at equilibrium (Eqn. 6=0):

$$(\delta V_{12}B_1 - a\hat{R}_3) - V_{33}B_3 = 0 \tag{13}$$

which yields:

$$B_3 = \delta\frac{V_{12}}{V_{33}}B_1 - \frac{a\hat{R}_3}{V_{33}} = \delta\frac{y_{33}\mu_{12}\hat{R}_2}{y_{12}\mu_{33}\hat{R}_3}B_1 - \frac{ay_{33}}{\mu_{33}}. \tag{14}$$

The formula indicates that the density of species 3 is determined by the density of species 1 and the ratio of the densities of resource 2 and 3 at equilibrium. Further substituting Eqn.9-12 in Eqn. 14 yields

$$B_3 = \delta\frac{y_{33}}{y_{12}}B_1 - a\frac{y_{33}}{\mu_{33}} \tag{15}$$

$$\text{or } \frac{B_3}{B_1} = \delta\frac{y_{33}}{y_{12}} - a\frac{y_{33}}{\mu_{33}}\frac{1}{B_1}. \tag{16}$$

The ratio indicates that there is a minimum requirement for the density of species 1 for species 3 to sustain, i.e.,

$$B_1 = \frac{a}{\delta}\frac{y_{12}}{\mu_{33}}. \tag{17}$$

With the increase of the supply of resources and B_1, the ratio tends to converge to a fixed value,

$$\frac{B_3}{B_1} \to \delta\frac{y_{33}}{y_{12}}. \tag{18}$$

Then, we can further derive how much change would be made to the consumption vector when consider species 1 and 3 as a cohort.

We can rewrite the dynamics of resource 1 (Eqn. 4) as

$$\begin{aligned}\frac{dR_1}{dt} &= a(s_1 - R_1) - V_{11}N_1 - V_{21}N_2 - V_{31}N_3 \\ &= a(s_1 - R_1) - V_{11}N_1 - V_{31}\delta\frac{y_{33}}{y_{12}}N_1 - V_{21}N_2 \\ &= a(s_1 - R_1) - \left(\frac{\mu_{12}R_2}{y_{11}} + \delta\frac{y_{33}}{y_{12}}\frac{\mu_{33}R_3}{y_{31}}\right)N_1 - V_{21}N_2\end{aligned}$$

Thus, we have

$$\frac{dR_1}{dt} = a(s_1 - R_1) - (V_{11} + V_{31}\delta\frac{y_{33}}{y_{12}})N_1 - V_{21}N_2 \tag{19}$$

$$\frac{dR_2}{dt} = a(s_2 - R_2) - V_{12}N_1 - V_{22}N_2. \tag{20}$$

From these equations, we know that the consumption vector of the team of species 1 and 3 is changed. The team has a higher impact on resource 1 than species 1 alone. Specifically, the slope of the consumption vector of the cohort on resources is given by

$$
\begin{aligned}
c_{13} &= \frac{V_{12}}{V_{11} + V_{31}\delta \frac{y_{33}}{y_{12}}} \\
&= \frac{\frac{\mu_{12}}{y_{12}} R_2}{\left(\frac{\mu_{12} R_2}{y_{11}} + \delta \frac{y_{33}}{y_{12}} \frac{\mu_{33} R_3}{y_{31}}\right)} \\
&= \frac{1/y_{12}}{\left(1/y_{11} + \delta \frac{1}{y_{12}} \frac{y_{33}}{y_{31}}\right)} = \frac{y_{11}}{y_{12}} / \left(1 + \delta \frac{y_{11}}{y_{12}} \frac{y_{33}}{y_{31}}\right) \qquad (21)
\end{aligned}
$$

while when there is no recipient group, the slope is given by

$$c_1 = \frac{1/y_{12}}{1/y_{11}} = \frac{y_{11}}{y_{12}}. \qquad (22)$$

So, with the dependence, the slope becomes shallower, which narrows the coexistence area with species 2.

2 Deriving the Requirement of the Density of Species 1 for the Recipient Species to Persist

2.1 Species 1 and Species 3

We first consider a scenario that there is only species 1 and explore when the recipient species can persist. Given the model, there are two cases. One is that when resource 1 is sufficiently supplied, species 1 is limited by resource 2, i.e., $s_2/s_1 < y_{11}/y_{12}$. Thus, we have

$$\frac{1}{B_1}\frac{dB_1}{dt} = \mu_{12} R_2 - m \qquad (23)$$

$$\frac{dR_2}{dt} = a(s_2 - R_2) - V_{12} B_1 \qquad (24)$$

which yields the equilibrium at

$$\hat{B}_1 = \frac{a}{m} s_2 y_{12} - a \frac{y_{12}}{\mu_{12}} \qquad (25)$$

$$\hat{R}_2 = \frac{m}{\mu_{12}}. \qquad (26)$$

From Eqn.17, there is a minimum requirement for the density of species 1 for the recipient species to persist. Thus, we have

$$\hat{B}_1 > \frac{a}{\delta} \frac{y_{12}}{\mu_{33}} \qquad (27)$$

which yields

$$s_2 > \frac{m}{\mu_{12}} + \frac{m}{\delta} \frac{1}{\mu_{33}} := s_2'. \qquad (28)$$

This indicates that when species 1 is limited by resource 2, the supply of resource 2 should be larger than the threshold such that the recipient species can persist. The point s' is given by

$$s' = (s_1', s_2') = \left(\frac{m}{\mu_{11}} + \frac{y_{12}}{y_{11}} \frac{m}{\delta} \frac{1}{\mu_{33}}, \frac{m}{\mu_{12}} + \frac{m}{\delta} \frac{1}{\mu_{33}}\right) \qquad (29)$$

Similarly, we can derive the criteria for the supply rate of resource 1 when species 1 is limited by resource 1, i.e., $s_2/s_1 > y_{11}/y_{12}$, yielding

$$s_1 > \frac{m}{\mu_{11}} + \frac{m}{\delta} \frac{y_{12}}{y_{11}} \frac{1}{\mu_{33}} := s_1'. \qquad (30)$$

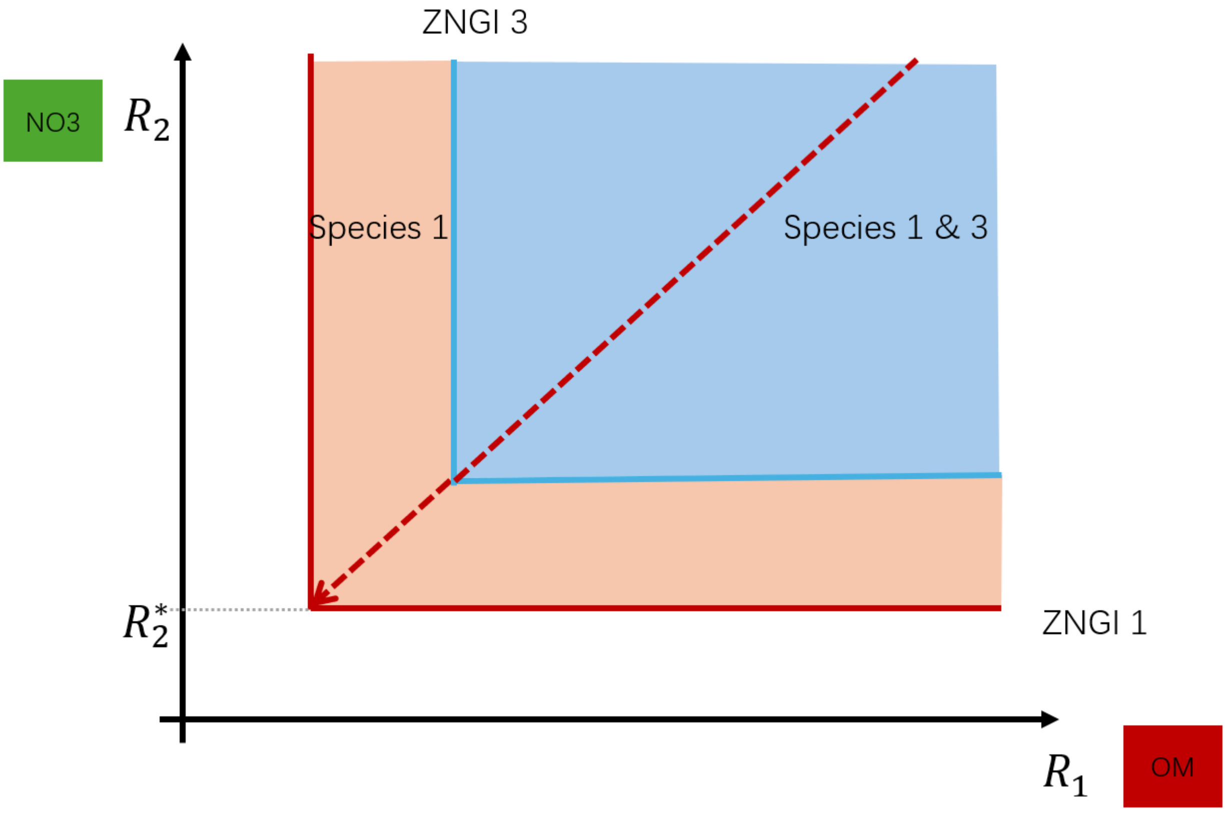

Figure 1: Graphic analysis of a system with species 1 and 3.

The point s'' is given by

$$s'' = (s_1'', s_2'') = (\frac{m}{\mu_{11}} + \frac{m}{\delta}\frac{y_{12}}{y_{11}}\frac{1}{\mu_{33}}, \frac{m}{\mu_{12}} + \frac{m}{\delta}\frac{1}{\mu_{33}}) \tag{31}$$

Thus, the two points s' and s'' are identical to each other.

So, we have the resource plane divided into 3 regions. Below the ZNGI of species 1, no species can sustain. When the supply of resources are intermediate, only species 1 can sustain. When the supply is sufficient, the recipient species can coexist with species 1.

2.2 Species 1, Species 2 and Species 3

We consider the coexistence of species 1 and 2 at equilibrium

$$a(s_1 - R_1) - V_{11}B_1 - V_{21}B_2 = 0 \tag{32}$$

$$a(s_2 - R_2) - V_{12}B_1 - V_{22}B_2 = 0 \tag{33}$$

$$\hat{R}_1 = \frac{m}{\mu_{21}}, \hat{R}_2 = \frac{m}{\mu_{12}} \tag{34}$$

We can solve for the densities of the species as

$$\hat{B}_1 = \frac{\frac{a}{m}\left((\frac{y_{21}}{y_{22}}s_1 - s_2) + \frac{m}{\mu_{12}} - \frac{y_{21}}{y_{22}}\frac{m}{\mu_{21}}\right)}{\frac{y_{21}}{y_{22}}\frac{1}{y_{11}} - \frac{1}{y_{12}}} \tag{35}$$

$$\hat{B}_2 = \frac{\frac{a}{m}\left(\frac{y_{11}}{y_{12}}s_1 - s_2 - \frac{y_{11}}{y_{12}}\frac{m}{\mu_{21}} + \frac{m}{\mu_{12}}\right)}{\frac{y_{11}}{y_{12}}\frac{1}{y_{21}} - \frac{1}{y_{22}}} \tag{36}$$

The minimum requirement for the density of species 1 is given by Eqn. 17. Thus, we can obtain the boundary on the resource plane where species 3 can invade, yielding

$$\frac{\frac{a}{m}\left((\frac{y_{21}}{y_{22}}s_1 - s_2) + \frac{m}{\mu_{12}} - \frac{y_{21}}{y_{22}}\frac{m}{\mu_{21}}\right)}{\frac{y_{21}}{y_{22}}\frac{1}{y_{11}} - \frac{1}{y_{12}}} > \frac{a}{\delta}\frac{y_{12}}{\mu_{33}} \tag{37}$$

which leads to

$$s_2 < \frac{y_{21}}{y_{22}}s_1 + \frac{m}{\mu_{12}} - \frac{y_{21}}{y_{22}}\frac{m}{\mu_{21}} + \frac{1}{\delta}\left(\frac{y_{11}y_{22} - y_{12}y_{21}}{y_{11}y_{22}}\right)\frac{m}{\mu_{33}} := c_{12}. \tag{38}$$

When species 2 invades a community of species 1 and 3 coexisting, the equilibrium state of species 1 and 3 and resources is given by

$$\hat{R}_2 = \frac{m}{\mu_{12}}, \hat{R}_3 = \frac{m}{\mu_{33}} \tag{39}$$

$$V_{11}B_1 + V_{31}B_3 = a(s_1 - \hat{R}_1) \tag{40}$$

$$V_{12}B_1 = a(s_2 - \hat{R}_2) \tag{41}$$

$$\delta V_{12}B_1 = a\hat{R}_3 + V_{33}B_3 \tag{42}$$

which leads to

$$\begin{aligned}\hat{R}_1 &= s_1 - \frac{m}{a}\frac{1}{y_{11}}B_1 - \frac{m}{a}\frac{1}{y_{31}}B_3 \\ &= s_1 - (\frac{y_{12}}{y_{11}} + \delta\frac{y_{33}}{y_{31}})s_2 + (\frac{y_{12}}{y_{11}} + \delta\frac{y_{33}}{y_{31}})\frac{m}{\mu_{12}} + \frac{y_{33}}{y_{31}}\frac{m}{\mu_{33}}.\end{aligned} \tag{43}$$

Now, we can assess the invasion growth rate of species 2:

$$G_2 = \mu_{21}\hat{R}_1 - m > 0 \tag{44}$$

which leads to the boundary that species 2 can invade

$$s_2 = \Delta_2 s_1 - \Delta_2\frac{m}{\mu_{21}} + \Delta_2\frac{y_{33}}{y_{31}}\frac{m}{\mu_{33}} + \frac{m}{\mu_{12}} \tag{45}$$

where

$$\Delta_2 = \frac{\frac{y_{11}}{y_{12}}}{1 + \delta\frac{y_{11}}{y_{12}}\frac{y_{33}}{y_{31}}}$$

Then, the crossing point B is where the two boundaries intersect, yielding

$$s_1' = \frac{m}{\mu_{21}} + \frac{\Delta_2\frac{y_{33}}{y_{31}} - \frac{1}{\delta}(\frac{y_{11}y_{22} - y_{12}y_{21}}{y_{11}y_{22}})}{\frac{y_{21}}{y_{22}} - \Delta_2}\frac{m}{\mu_{33}} \tag{46}$$

$$s_2' = \Delta_2 s_1' - \Delta_2\frac{m}{\mu_{21}} + \Delta_2\frac{y_{33}}{y_{31}}\frac{m}{\mu_{33}} + \frac{m}{\mu_{12}} \tag{47}$$

2.3 Species 1, 3 and 5

Similarly, we can compute the threshold for the existence of species 5. As species 5 is not competing for either resource 1 or 2. It does not affect the coexistence of species 1 and 2. Thus, we only need to calculate when species 5 can exist when species 1 can produce sufficient resource 3 for it. Here, we assume that species

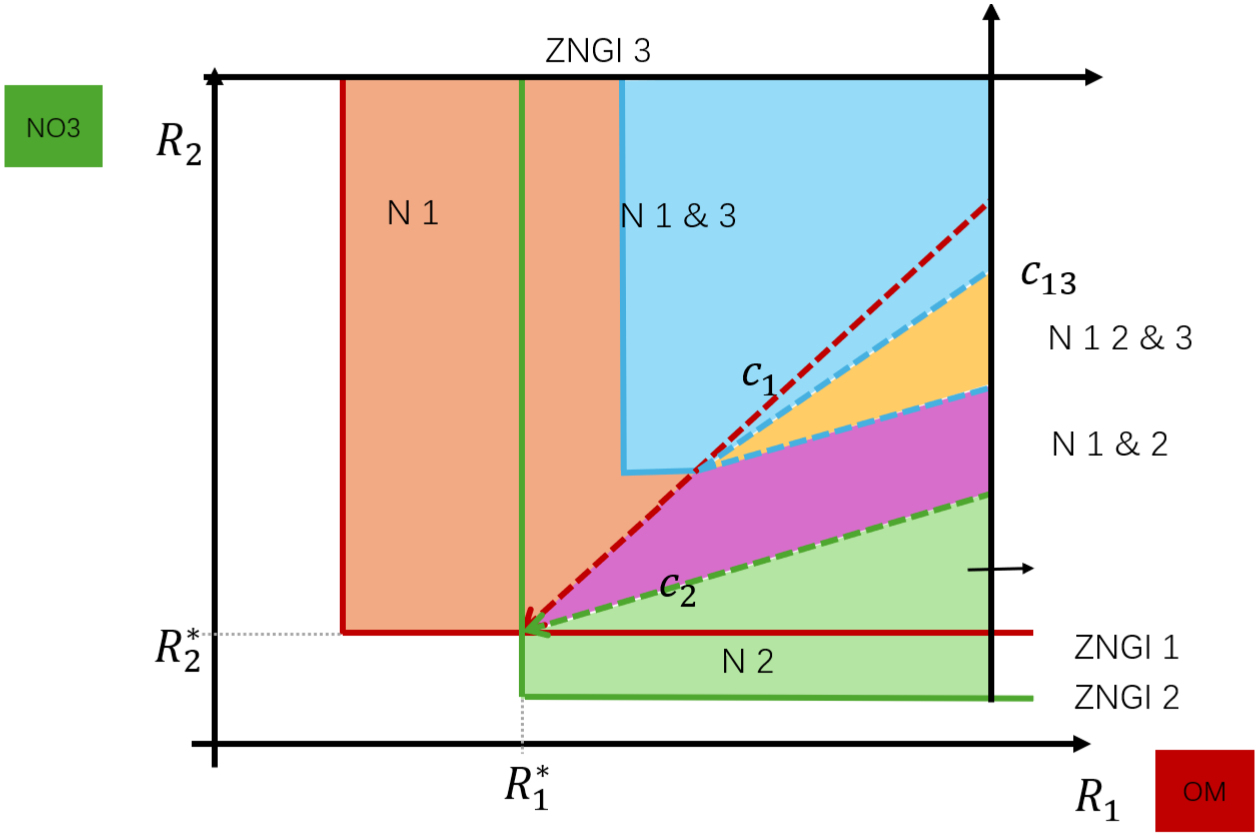

Figure 2: Graphical analysis of species 1, species 2, species 3.

5 is limited by resource 5. The system is given by

$$\frac{1}{N_1}\frac{dB_1}{dt} = \min(\mu_{11}R_1, \mu_{12}R_2) - m \tag{48}$$

$$\frac{1}{B_3}\frac{dB_3}{dt} = \min(\mu_{31}R_1, \mu_{33}R_3) - m \tag{49}$$

$$\frac{1}{B_5}\frac{dB_5}{dt} = \min(\mu_{53}R_3, \mu_{55}R_5) - m \tag{50}$$

$$\frac{dR_1}{dt} = a(s_1 - R_1) - V_{11}B_1 - V_{31}B_3 \tag{51}$$

$$\frac{dR_2}{dt} = a(s_2 - R_2) - V_{12}B_1 \tag{52}$$

$$\frac{dR_3}{dt} = (\delta V_{12}B_1 - aR_3) - V_{33}B_3 - V_{53}B_5 \tag{53}$$

$$\frac{dR_5}{dt} = (s_5 - aR_5) - V_{55}B_5. \tag{54}$$

The equilibrium state of the resources for these three species to coexist is given by

$$\hat{R}_1 = \frac{m}{\mu_{21}}, \hat{R}_2 = \frac{m}{\mu_{12}}, \hat{R}_3 = \frac{m}{\mu_{33}}, \hat{R}_5 = \frac{m}{\mu_{55}}. \tag{55}$$

From Eqn. 53, we know the requirement of the density of species 1 for the density of species 3 to be positive, given by

$$\delta V_{12}\hat{B}_1 - aR_3 - V_{53}\hat{B}_5 > 0 \tag{56}$$

leading to

$$\hat{B}_1 > \frac{a}{\delta}\frac{y_{12}}{\mu_{33}} + \frac{ay_{12}}{\delta m}s_5 - \frac{a}{\delta}\frac{y_{12}}{\mu_{55}}. \tag{57}$$

If species 1 is limited by resource 2, the density is given by

$$\hat{B}_1 = \frac{a}{m}y_{12}s_2 - a\frac{y_{12}}{\mu_{12}} \tag{58}$$

Thus, we have the boundary

$$s_2 = \frac{m}{\delta\mu_{33}} + \frac{1}{\delta}(s_5 - \frac{m}{\mu_{55}}) + \frac{m}{\mu_{12}}. \tag{59}$$

If species 1 is limited by resource 1, the density is given by

$$\hat{B}_1 = \frac{a}{m}y_{11}s_1 - a\frac{y_{11}}{\mu_{11}}. \tag{60}$$

Thus, the boundary is given by

$$s_2 = \frac{m}{\mu_{11}} + \frac{m}{\delta\mu_{33}}\frac{y_{12}}{y_{11}} + \frac{1}{\delta}\frac{y_{12}}{y_{11}}(s_5 - \frac{m}{\mu_{55}}). \tag{61}$$

Compared to the system of only species 1 and 3 (Eqn. 30), an additional amount of supply is needed due to the presence of species 5. The amount is determined by $\frac{1}{\delta}\frac{y_{12}}{y_{11}}(s_5 - \frac{m}{\mu_{55}})$, which depends on the supply of resource 5 and the trait of species 5.

2.4 Species 1, 2, 3 and 5

Here, we consider a system of species 1, 2, 3 and 5. As species 5 is a better competitor for resource 3, it has to be limited by resource 5 for species 3 to persist. Thus, the system is described as

$$\frac{1}{B_1}\frac{dB_1}{dt} = \min(\mu_{11}R_1, \mu_{12}R_2) - m \tag{62}$$
$$\frac{1}{B_2}\frac{dB_2}{dt} = \min(\mu_{21}R_1, \mu_{22}R_2) - m \tag{63}$$
$$\frac{1}{B_3}\frac{dB_3}{dt} = \min(\mu_{31}R_1, \mu_{33}R_3) - m \tag{64}$$
$$\frac{1}{B_5}\frac{dB_5}{dt} = \min(\mu_{53}R_3, \mu_{55}R_5) - m \tag{65}$$
$$\frac{dR_1}{dt} = a(s_1 - R_1) - V_{11}B_1 - V_{21}B_2 - V_{31}B_3 \tag{66}$$
$$\frac{dR_2}{dt} = a(s_2 - R_2) - V_{12}B_1 - V_{22}B_2 \tag{67}$$
$$\frac{dR_3}{dt} = (\delta V_{12}B_1 - aR_3) - V_{33}B_3 - V_{53}B_5 \tag{68}$$
$$\frac{dR_5}{dt} = (s_5 - aR_5) - V_{55}B_5. \tag{69}$$

As analyzed above, the equilibrium state of the resources for these four species to coexist is given by

$$\hat{R}_1 = \frac{m}{\mu_{21}}, \hat{R}_2 = \frac{m}{\mu_{12}}, \hat{R}_3 = \frac{m}{\mu_{33}}, \hat{R}_5 = \frac{m}{\mu_{55}} \tag{70}$$

We first consider what impact of the addition of species 5 to the system of species 1 and 3. From Eqn.68, we have

$$\delta V_{12}N_1 = aR_3 + V_{33}N_3 + V_{53}N_5. \tag{71}$$

Given the equilibrium condition for species 5

$$\hat{B}_5 = \frac{a}{m}s_5 y_{55} - a\frac{y_{55}}{\mu_{55}} \tag{72}$$

we obtain the impact of species 5 on species 3:

$$\begin{aligned}\hat{B}_3 &= \delta\frac{y_{33}}{y_{12}}\hat{B}_1 - a\frac{y_{33}}{\mu_{33}} - \frac{y_{53}}{y_{33}}\hat{B}_5 \\ &= \delta\frac{y_{33}}{y_{12}}(\frac{a}{m}s_2 y_{12} - a\frac{y_{12}}{\mu_{12}}) \\ &\quad - a\frac{y_{33}}{\mu_{33}} - \frac{y_{53}}{y_{33}}(\frac{a}{m}s_5 y_{55} - a\frac{y_{55}}{\mu_{55}}).\end{aligned} \tag{73}$$

This result indicates that species 5 will decrease the density of specie 3 by taking up its limiting resource, resource 3. Increasing the supply of resource 5 will further decrease the density of species 3.

We now examine the invasion of species 2 by assessing the invasion growth rate

$$G_2 = \mu_{21}\hat{R}_1 - m. \tag{74}$$

A positive invasion growth rate ensures existence of species 2, thus we should have

$$\hat{R}_1 > \frac{m}{\mu_{21}}. \tag{75}$$

Given that $\hat{R}_1 = s_1 - \frac{V_{11}}{a}\hat{B}_1 - \frac{V_{31}}{a}\hat{B}_3$ and $\hat{B}_1$ is given by Equn. 67 when $\hat{B}_2 = 0$, yielding

$$\hat{B}_1 = \frac{a}{m}s_2 y_{12} - a\frac{y_{12}}{\mu_{12}} \tag{76}$$

we obtain the inequality

$$s_1 - \frac{V_{11}}{a}\hat{B}_1 - \frac{V_{31}}{a}\hat{B}_3 > \frac{m}{\mu_{21}} \tag{77}$$

which leads to the expression for the boundary where species 2 can invade the community of species 1, 3 and 5

$$s_2 = \Delta_2 s_1 - \Delta_2\frac{m}{\mu_{21}} + \Delta_2\frac{y_{33}}{y_{31}}\frac{m}{\mu_{33}} + \frac{m}{\mu_{12}} + \Delta_2\frac{y_{53}}{y_{31}}\frac{y_{55}}{y_{33}}(s_5 - \frac{m}{\mu_{55}}). \tag{78}$$

Comparing Eqn. 78 and 45, we know that the addition of species 5 will elevate the boundary, which narrows species 3's niche.

Similarly, we can assess the invasion of species 3 to a community of species 1, 2 and 5. As species 5 has no impact on the interaction of species 1 and 2, the equilibrium of a system with species 1 and 2 and a system with species 1, 2 and 5 is the same for resources 1 and 2. So, we have the equilibrium density of species 1 given by Eqn. 35

$$\hat{B}_1 = \frac{\frac{a}{m}\left((\frac{y_{21}}{y_{22}}s_1 - s_2) + \frac{m}{\mu_{12}} - \frac{y_{21}}{y_{22}}\frac{m}{\mu_{21}}\right)}{\frac{y_{21}}{y_{22}}\frac{1}{y_{11}} - \frac{1}{y_{12}}}$$

and the equilibrium density of species 5 given by Eqn.72. The existence of species 3 requires its invasion growth rate to be positive,

$$G_3 = \mu_{33}\hat{R}_3 - m > 0. \tag{79}$$

From Eqn. 68, when species 3 is rare $\hat{B}_3 = 0$, the density of resource 3 is given by

$$\begin{aligned}\hat{R}_3 &= \frac{\delta}{a}V_{12}\hat{B}_1 - \frac{V_{53}}{a}\hat{B}_5 \\ &= \frac{\delta}{a}\frac{m}{y_{12}}\frac{\frac{a}{m}\left((\frac{y_{21}}{y_{22}}s_1 - s_2) + \frac{m}{\mu_{12}} - \frac{y_{21}}{y_{22}}\frac{m}{\mu_{21}}\right)}{\frac{y_{21}}{y_{22}}\frac{1}{y_{11}} - \frac{1}{y_{12}}} \\ &- \frac{m}{ay_{53}}(\frac{a}{m}s_5 y_{55} - a\frac{y_{55}}{\mu_{55}}) > \frac{m}{\mu_{33}}\end{aligned} \tag{80}$$

which yields the boundary

$$\begin{aligned}s_2 = &\frac{y_{21}}{y_{22}}s_1 + \frac{m}{\mu_{12}} - \frac{y_{21}}{y_{22}}\frac{m}{\mu_{21}} + \frac{1}{\delta}\left(\frac{y_{11}y_{22} - y_{12}y_{21}}{y_{11}y_{22}}\right)\frac{m}{\mu_{33}} \\ &+ \frac{1}{\delta}\frac{y_{11}y_{22} - y_{12}y_{21}}{y_{11}y_{22}}(s_5 - \frac{m}{\mu_{55}})\frac{y_{55}}{y_{53}}\end{aligned} \tag{81}$$

Then, the crossing point B is where the two boundaries intersect, yielding

$$s_1' = \frac{m}{\mu_{21}} + \frac{\Delta_2\frac{y_{33}}{y_{31}} - \frac{1}{\delta}(\frac{y_{11}y_{22}-y_{12}y_{21}}{y_{11}y_{22}})}{\frac{y_{21}}{y_{22}} - \Delta_2}\frac{m}{\mu_{33}} + (\frac{1}{\delta}\frac{y_{11}y_{22} - y_{12}y_{21}}{y_{11}y_{22}}\frac{y_{55}}{y_{53}} - \Delta_2\frac{y_{53}}{y_{31}}\frac{y_{55}}{y_{33}})(s_5 - \frac{m}{\mu_{55}}) \tag{82}$$

$$s_2' = \Delta_2 s_1' - \Delta_2\frac{m}{\mu_{21}} + \Delta_2\frac{y_{33}}{y_{31}}\frac{m}{\mu_{33}} + \frac{m}{\mu_{12}} + \Delta_2\frac{y_{53}}{y_{31}}\frac{y_{55}}{y_{33}}(s_5 - \frac{m}{\mu_{55}}) \tag{83}$$

2.5 Species 2 and species 4

Similarly, we consider the condition for coexistence of species 2 and species 4.

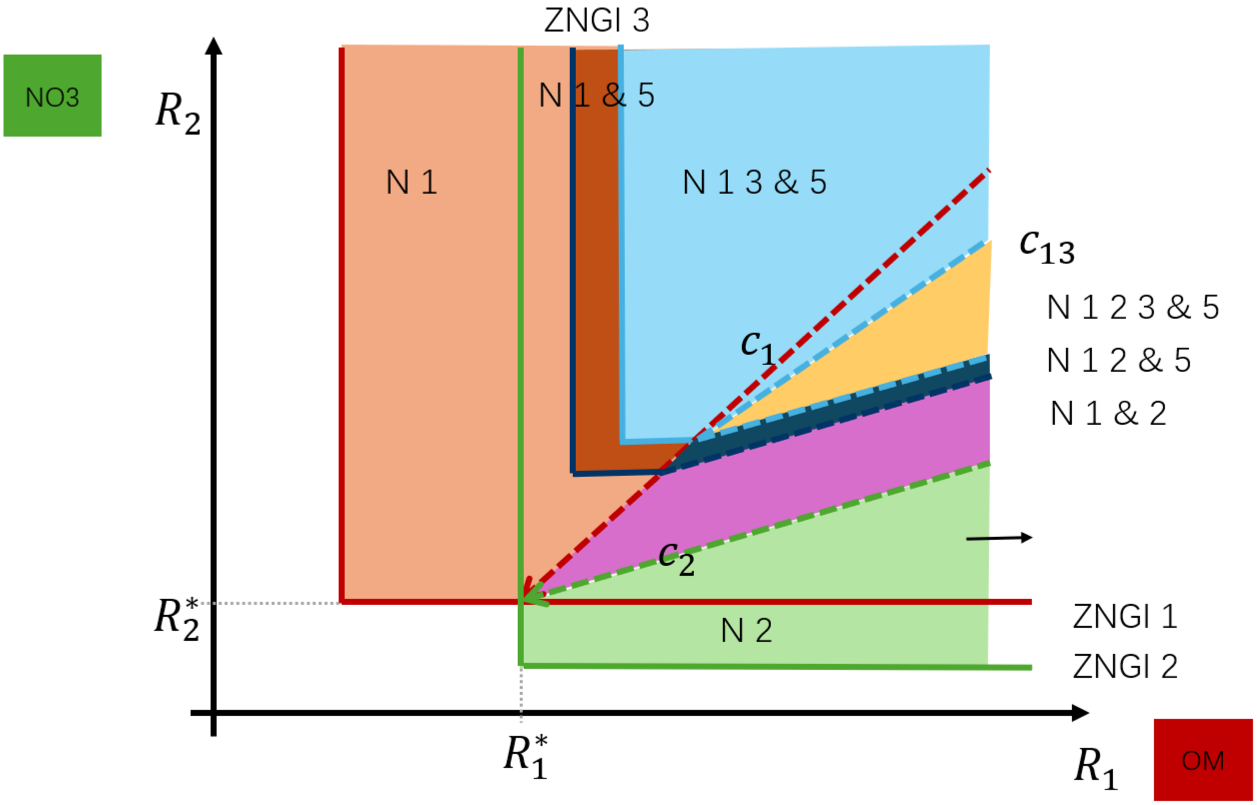

Figure 3: Graphical analysis of species 1, species 2, species 3 and species 5.

$$\frac{1}{B_2}\frac{dB_2}{dt} = \min(\mu_{21}R_1, \mu_{22}R_2) - m \tag{84}$$

$$\frac{1}{B_4}\frac{dB_4}{dt} = \min(\mu_{41}R_1, \mu_{44}R_4) - m \tag{85}$$

$$\frac{dR_1}{dt} = a(s_1 - R_1) - V_{21}B_2 - V_{41}B_4 \tag{86}$$

$$\frac{dR_2}{dt} = a(s_2 - R_2) - V_{22}B_2 \tag{87}$$

$$\frac{dR_4}{dt} = (s_{24}(B_2) - aR_4) - V_{44}B_4. \tag{88}$$

Again, we assume that the production of resource 4 is proportional to the uptake of resource 2 via the conversion by species 2, given by $s_{24}(B_2) = \delta V_{22}B_2$.

Species 4 can be limited either by resource 1 or resource 4. This depends on which resource is the limiting factor for species 2. If species 2 is limited by resource 1, species 4 should have a lower R^*_{41} than that of species 2, R^*_{21}. Otherwise, species 4 cannot persist as resource 1 would always be drawn down to the R^*_{21} level of species 2. However, if species 2 is limited by resource 2, it is possible for species having a larger R^*_{41} on resource 1 than species 2 to exist, as the equilibrium density of resource 1 can exceed the R^* of both species on resource 1.

In our chosen system, species 2 is a weaker competitor for resource 1. Thus, we can assume that $\mu_{41} > \mu_{21}$ and species 4 is majorly limited by resource 4. The density of resource 4 at equilibrium is then given by $R_4 = \frac{m}{\mu_{44}}$. From Eqn. 88, we can solve for the minimum requirement of the density of species 2

$$B_4 = \frac{\delta V_{22}B_2 - a\frac{m}{\mu_{44}}}{V_{44}} > 0 \tag{89}$$

which leads to

$$B_2 > \frac{a}{\delta}\frac{y_{22}}{\mu_{44}}. \tag{90}$$

Using the equilibrium state for Eqn. 88 (setting Eqn. 88=0), the relationship between the densities of species 2 and 4 is

$$B_4 = \delta\frac{y_{44}}{y_{22}}B_2 - a\frac{y_{44}}{\mu_{44}} \tag{91}$$

This equation can be used to determine the consumption vector on the resource plane of resource 1 and 2 for the team of species 2 and 4. We define $B_{24} = B_2 + B_4$. Then,

$$B_2 = \frac{y_{22}}{y_{22} + \delta y_{44}}B_{24} + a\frac{y_{22}}{y_{22} + \delta y_{44}}\frac{y_{44}}{\mu_{44}} \tag{92}$$

$$B_4 = \frac{\delta y_{44}}{y_{22} + \delta y_{44}}B_{24} - a\frac{y_{22}}{y_{22} + \delta y_{44}}\frac{y_{44}}{\mu_{44}} \tag{93}$$

The dynamics of resource 1 and 2 can be rewritten as

$$\frac{dR_1}{dt} = a(s_1 - R_1) - (V_{21}\frac{y_{22}}{y_{22} + \delta y_{44}} + V_{41}\frac{\delta y_{44}}{y_{22} + \delta y_{44}})B_{24} - (a\frac{y_{22}}{y_{22} + \delta y_{44}}\frac{y_{44}}{\mu_{44}}V_{21} - a\frac{y_{22}}{y_{22} + \delta y_{44}}\frac{y_{44}}{\mu_{44}}V_{41}) \tag{94}$$

$$\frac{dR_2}{dt} = a(s_2 - R_2) - V_{22}\frac{y_{22}}{y_{22} + \delta y_{44}}B_{24} - a\frac{y_{22}}{y_{22} + \delta y_{44}}\frac{y_{44}}{\mu_{44}}V_{22}. \tag{95}$$

We obtain the consumption vector of the team of species 2 and 4 on the resource plane of resource 1 and 2 as

$$c_{24} = \frac{V_{22}\frac{y_{22}}{y_{22}+\delta y_{44}}}{V_{21}\frac{y_{22}}{y_{22}+\delta y_{44}} + V_{41}\frac{\delta y_{44}}{y_{22}+\delta y_{44}}} = \frac{y_{21}}{y_{22}}/(1 + \delta\frac{y_{44}}{y_{41}}\frac{y_{21}}{y_{22}}). \tag{96}$$

This consumption vector indicates that the team of species 2 and 4 has a shallower slope than species 2.

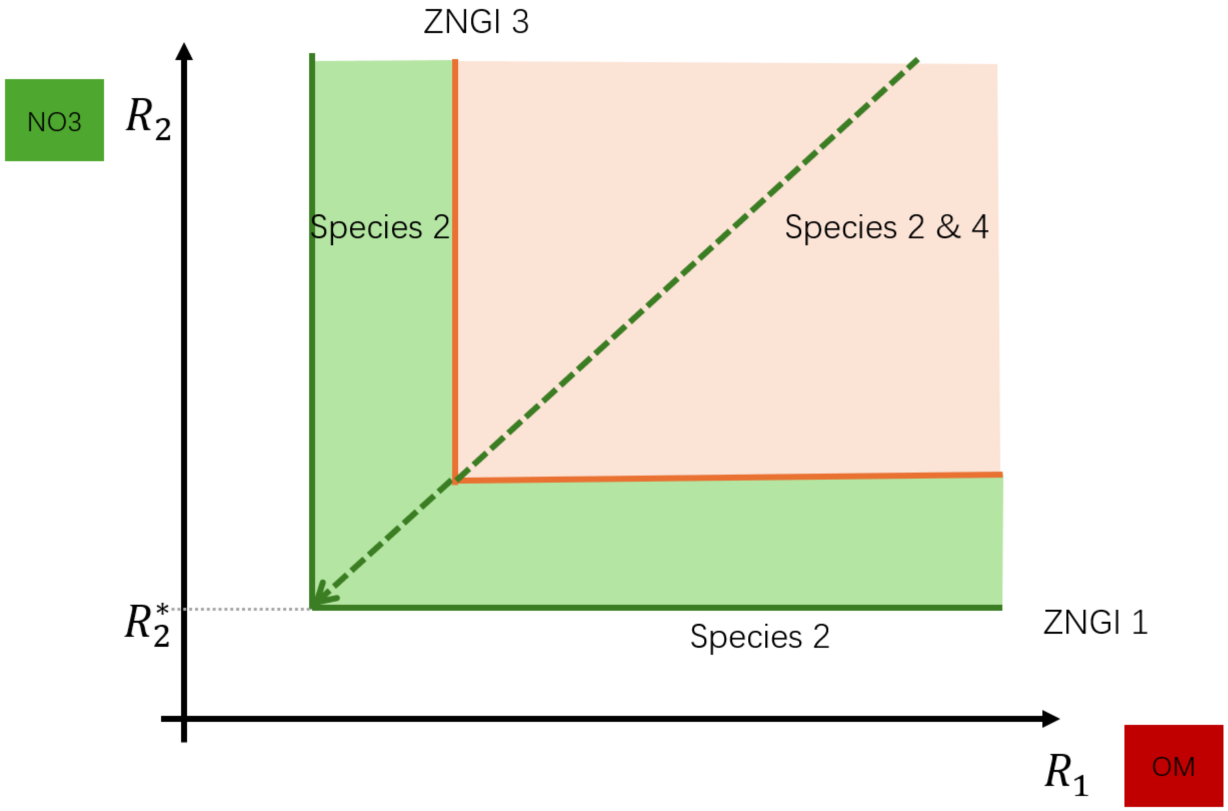

Figure 4: Graphical analysis of species 2 and 4.

2.6 Species 1, 2, 4

For the coexistence region for species 1, 2, and 4, we need to examine the boundary when species 1 invades a community where species 2 and 4 coexist at equilibrium. The other boundary is when species 4 invades a community where species 1 and 2 coexist at equilibrium.

To solve for the first boundary, we study the equilibrium state where species 2 and 4 are at equilibrium. The equilibrium state is given by

$$\hat{R}_1 = \frac{m}{\mu_{21}}, \hat{R}_4 = \frac{m}{\mu_{44}}, \hat{R}_2 = s_2 - \frac{m}{a y_{22}} \hat{B}_2 \tag{97}$$

$$\hat{B}_2 = \frac{a s_1 - am/\mu_{21} + am \frac{y_{44}}{y_{41}} \frac{1}{\mu_{44}}}{m/y_{21} + \delta m \frac{y_{44}}{y_{41}} \frac{1}{y_{22}}}. \tag{98}$$

As species 1 has to be limited by resource 2, the invasion growth rate of species 1 is

$$\begin{aligned} G_1 &= \mu_{12} \hat{R}_2 - m \\ &= \mu_{12} s_2 - \frac{m \mu_{12}}{a y_{22}} \frac{a s_1 - am/\mu_{21} + am \frac{y_{44}}{y_{41}} \frac{1}{\mu_{44}}}{m/y_{21} + \delta m \frac{y_{44}}{y_{41}} \frac{1}{y_{22}}} - m > 0 \end{aligned} \tag{99}$$

which yields

$$s_2 > \frac{\frac{y_{21}}{y_{22}}}{1 + \delta \frac{y_{44}}{y_{41}} \frac{y_{21}}{y_{22}}} s_1 - \frac{\frac{y_{21}}{y_{22}}}{1 + \delta \frac{y_{44}}{y_{41}} \frac{y_{21}}{y_{22}}} \frac{m}{\mu_{21}} + \frac{\frac{y_{21}}{y_{22}}}{1 + \delta \frac{y_{44}}{y_{41}} \frac{y_{21}}{y_{22}}} \frac{y_{44}}{y_{41}} \frac{m}{\mu_{44}} + \frac{m}{\mu_{12}} \tag{100}$$

The second boundary is at the equilibrium steady state where species 1 and 2 are at equilibrium.

$$\frac{1}{B_1} \frac{dB_1}{dt} = \min(\mu_{11} R_1, \mu_{12} R_2) - m \tag{101}$$

$$\frac{1}{B_2} \frac{dB_2}{dt} = \min(\mu_{21} R_1, \mu_{22} R_2) - m \tag{102}$$

$$\frac{dR_1}{dt} = a(s_1 - R_1) - V_{11} B_1 - V_{21} B_2 \tag{103}$$

$$\frac{dR_2}{dt} = a(s_2 - R_2) - V_{12} B_1 - V_{22} B_2 \tag{104}$$

We assume that species 1 is a better explorer on resource 1 while species 2 is a better explorer on resource 2. Thus, at equilibrium, species 1 is limited by resource 2 and species 2 is limited by resource 2. We can solve for equilibrium densities for resources

$$\hat{R}_1 = \frac{m}{\mu_{21}}, \hat{R}_2 = \frac{m}{\mu_{12}} \tag{105}$$

and for species

$$\hat{B}_1 = \frac{\frac{a}{m} \left((\frac{y_{21}}{y_{22}} s_1 - s_2) + \frac{m}{\mu_{12}} - \frac{y_{21}}{y_{22}} \frac{m}{\mu_{21}} \right)}{\frac{y_{21}}{y_{22}} \frac{1}{y_{11}} - \frac{1}{y_{12}}} \tag{106}$$

$$\hat{B}_2 = \frac{\frac{a}{m} \left(\frac{y_{11}}{y_{12}} s_1 - s_2 - \frac{y_{11}}{y_{12}} \frac{m}{\mu_{21}} + \frac{m}{\mu_{12}} \right)}{\frac{y_{11}}{y_{12}} \frac{1}{y_{21}} - \frac{1}{y_{22}}}. \tag{107}$$

As we have a minimum requirement for the density of species 2 for species 4 to persist (Eqn. 90), we can solve for the second boundary

$$s_2 < \frac{y_{11}}{y_{12}} s_1 - \frac{y_{11}}{y_{12}} \frac{m}{\mu_{21}} + \frac{m}{\mu_{12}} - \frac{m}{\delta} \frac{1}{\mu_{44}} \left(\frac{y_{11} y_{22} - y_{12} y_{21}}{y_{12} y_{21}} \right) \tag{108}$$

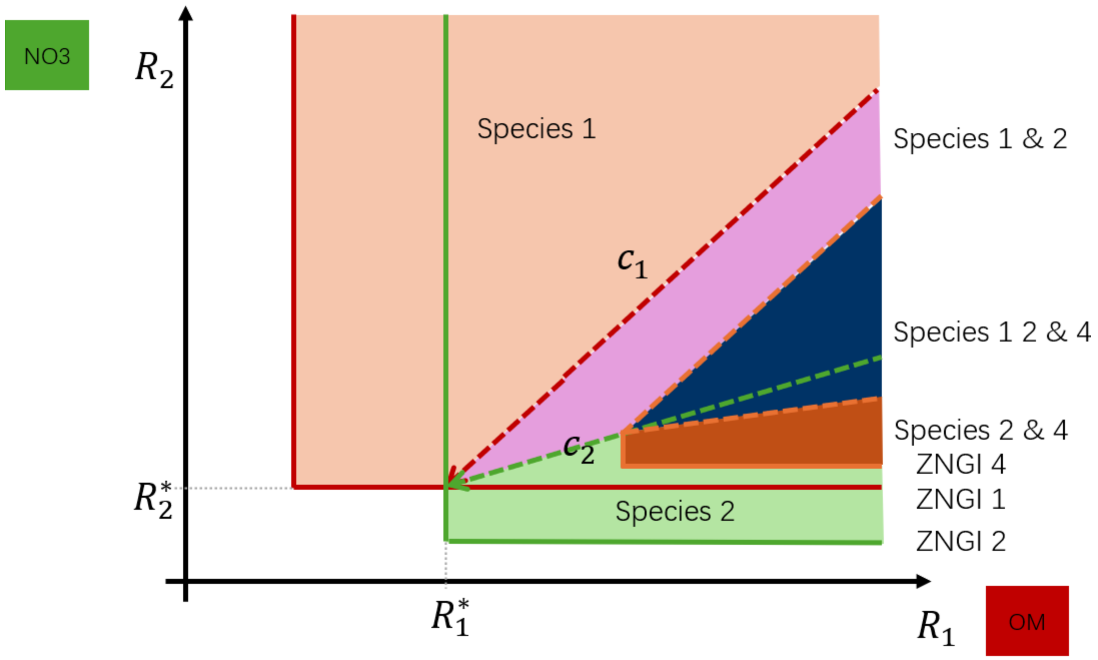

Figure 5: Graphical analysis of species 1, species 2, and species 4.

Thus, we obtain the crossing point of these two boundaries at

$$s_1' = \frac{m}{\mu_{21}} + \left(\frac{\Delta_1 \frac{y_{44}}{y_{41}} + \frac{1}{\delta} \frac{y_{11}y_{22} - y_{12}y_{21}}{y_{12}y_{21}}}{\frac{y_{11}}{y_{12}} - \Delta_1} \right) \frac{m}{\mu_{44}} \tag{109}$$

$$s_2' = \Delta_1 s_1' - \Delta_1 \frac{m}{\mu_{21}} + \Delta_1 \frac{y_{44}}{y_{41}} \frac{m}{\mu_{44}} + \frac{m}{\mu_{12}} \tag{110}$$

$$\Delta_1 = \frac{\frac{y_{21}}{y_{22}}}{1 + \delta \frac{y_{44}}{y_{41}} \frac{y_{21}}{y_{22}}} \tag{111}$$

2.7 Species 1, 2, 3, 4

From the analysis above, the coexistence region of the four species should be bounded by two boundaries. The two boundaries are determined by whether species 3 can invade a community where species 1, 2, 4 coexist and whether species 4 can invade a community where species 1, 2, 3 coexist. Thus, we can again apply invasion analysis to calculate the boundaries.

For the first case, the invasion growth rate of species 3 is given by

$$\lambda_3 = \mu_{33} \hat{R}_3 - m \tag{112}$$

because species 3 has to be limited by resource 3. Otherwise, if species 3 is limited by resource 1, given it is a better competitor for resource 1 than species 1 species 1 will be excluded. The concentration of resource 3 is determined by the density of its producer, species 1. From Eqn. 6, we know that there is a minimum requirement for the density of species 1 that can produce enough resource 3 for species 3 to persist (Eqn. 27). So, we need to calculate the density of species 1 at equilibrium in a community where species 1, 2, 4 can stably coexist.

The equilibrium is given by

$$\hat{R}_1 = \frac{m}{\mu_{21}}, \hat{R}_2 = \frac{m}{\mu_{12}}, \hat{R}_4 = \frac{m}{\mu_{44}} \tag{113}$$

and the densities of species are given by

$$a(s_1 - \hat{R}_1) = V_{11}\hat{B}_1 + V_{21}\hat{B}_2 + V_{41}\hat{B}_4 \tag{114}$$

$$a(s_2 - \hat{R}_2) = V_{12}\hat{B}_1 + V_{22}\hat{B}_2. \tag{115}$$

From Eqn. 91, we can replace $\hat{B}_4$ with $\delta\frac{y_{44}}{y_{22}}B_2 - a\frac{y_{44}}{\mu_{44}}$. Solving the equations leads to the density of species 1, given by

$$\hat{B}_1 = \frac{as_1 - a\hat{R}_1 + a\frac{m}{\mu_{44}}\frac{y_{44}}{y_{41}} - a(s_2 - \hat{R}_2)(\frac{y_{22}}{y_{21}} + \delta\frac{y_{44}}{y_{41}})}{\frac{m}{y_{11}} - \frac{m}{y_{12}}(\frac{y_{22}}{y_{21}} + \delta\frac{y_{44}}{y_{41}})}. \tag{116}$$

This density has to be larger than $\frac{a}{\delta}\frac{y_{12}}{\mu_{33}}$ for the persistence of species 3. Thus, we get the boundary for species 3 to invade

$$s_2 = \Delta_1 s_1 - \Delta_1\frac{m}{\mu_{21}} + \Delta_1\frac{m}{\mu_{44}}\frac{y_{44}}{y_{41}} - \Delta_1\frac{1}{\delta}\frac{m}{\mu_{33}}\frac{y_{12}}{y_{11}} + \frac{1}{\delta}\frac{m}{\mu_{33}} + \frac{m}{\mu_{12}} \tag{117}$$

where

$$\Delta_1 = \frac{1}{\frac{y_{22}}{y_{21}} + \delta\frac{y_{44}}{y_{41}}}. \tag{118}$$

Similarly, for the second case where species 4 invades a community of species 1, 2, 3 coexisting, we have the densities of species 1, 2, 3 at equilibrium

$$a(s_1 - \hat{R}_1) = V_{11}\hat{B}_1 + V_{21}\hat{B}_2 + V_{31}\hat{B}_3 \tag{119}$$

$$a(s_2 - \hat{R}_2) = V_{12}\hat{B}_1 + V_{22}\hat{B}_2. \tag{120}$$

with the resources at

$$\hat{R}_1 = \frac{m}{\mu_{21}}, \hat{R}_2 = \frac{m}{\mu_{12}}, \hat{R}_3 = \frac{m}{\mu_{33}}. \tag{121}$$

Again, at equilibrium there is a relationship between species 1 and species 3 (Eqn. 15), which can be used to replace $\hat{B}_3$ with $\delta\frac{y_{33}}{y_{12}}B_1 - a\frac{y_{33}}{\mu_{33}}$. This leads to the solution for the density of species 2, yielding

$$\hat{B}_2 = \frac{as_1 - a\hat{R}_1 + a\frac{m}{\mu_{33}}\frac{y_{33}}{y_{31}} - a(s_2 - \hat{R}_2)(\frac{y_{12}}{y_{11}} + \delta\frac{y_{33}}{y_{31}})}{\frac{m}{y_{21}} - \frac{m}{y_{22}}(\frac{y_{12}}{y_{11}} + \delta\frac{y_{33}}{y_{31}})} \tag{122}$$

For specie 4 to persist, there is a minimum requirement for the density of species 2 (Eqn. 90). Thus, we solve for the boundary

$$s_2 = \Delta_2 s_1 - \Delta_2\frac{m}{\mu_{21}} + \Delta_2\frac{m}{\mu_{33}}\frac{y_{33}}{y_{31}} - \Delta_2\frac{1}{\delta}\frac{m}{\mu_{44}}\frac{y_{22}}{y_{21}} + \frac{1}{\delta}\frac{m}{\mu_{44}} + \frac{m}{\mu_{12}} \tag{123}$$

where

$$\Delta_2 = \frac{1}{\frac{y_{12}}{y_{11}} + \delta\frac{y_{33}}{y_{31}}} \tag{124}$$

2.8 The Full System

For the full system that include all seven function groups, we can just use the boundaries derived above and merge the corresponding regions. This produces the Figure 4 in the main text.

3 How Much Ammonium is Needed to Make Anammox Limited by Nitrite

In this system, species 1 and 2 are the two primary feeder species while species 3 represents anammox. The growth follows Monod kinetics limited by the most scarce resource (Liebig's Law of the Minimum).

Microbial Biomass Equations:

$$\frac{dB_1}{dt} = B_1 \left(\min \left\{ \mu_{11} \frac{R_1}{R_1 + K_{11}}, \mu_{12} \frac{R_2}{R_2 + K_{12}} \right\} - a \right) \tag{125}$$

$$\frac{dB_2}{dt} = B_2 \left(\min \left\{ \mu_{21} \frac{R_1}{R_1 + K_{21}}, \mu_{22} \frac{R_2}{R_2 + K_{22}} \right\} - a \right) \tag{126}$$

$$\frac{dB_3}{dt} = B_3 \left(\min \left\{ \mu_{33} \frac{R_3}{R_3 + K_{33}}, \mu_{35} \frac{R_5}{R_5 + K_{35}} \right\} - a \right) \tag{127}$$

Substrate Concentration Equations:

$$\frac{dR_1}{dt} = a(s_1 - R_1) - \frac{\mu_1(R_1, R_2)}{y_{11}} B_1 - \frac{\mu_2(R_1, R_2)}{y_{21}} B_2 \tag{128}$$

$$\frac{dR_2}{dt} = a(s_2 - R_2) - \frac{\mu_1(R_1, R_2)}{y_{12}} B_1 - \frac{\mu_2(R_1, R_2)}{y_{22}} B_2 \tag{129}$$

$$\frac{dR_3}{dt} = B_1 - aR_3 - \frac{\mu_3(R_3, R_5)}{y_{33}} B_3 \tag{130}$$

$$\frac{dR_5}{dt} = a(xs_1 - R_5) - \frac{\mu_3(R_3, R_5)}{y_{35}} B_3 \tag{131}$$

Where μ_i represents the specific growth rate function for species i.

At steady state, the growth rate of each existing species must equal the dilution rate a. Based on the limitation conditions provided (B_1 limited by R_2, B_2 limited by R_1, and B_3 limited by R_3):

For B_1: $\mu_{12} \frac{R_2^*}{R_2^* + K_{12}} = a \implies R_2^* = \frac{aK_{12}}{\mu_{12} - a}$

For B_2: $\mu_{21} \frac{R_1^*}{R_1^* + K_{21}} = a \implies R_1^* = \frac{aK_{21}}{\mu_{21} - a}$

For B_3: $\mu_{33} \frac{R_3^*}{R_3^* + K_{33}} = a \implies R_3^* = \frac{aK_{33}}{\mu_{33} - a}$

Solving for Biomass B_1: The mass balance equations for R_1 and R_2 at steady state are:

$a(s_1 - R_1^*) - \frac{a}{y_{11}} B_1^* - \frac{a}{y_{21}} B_2^* = 0 \implies \frac{B_1^*}{y_{11}} + \frac{B_2^*}{y_{21}} = s_1 - R_1^*$

$a(s_2 - R_2^*) - \frac{a}{y_{12}} B_1^* - \frac{a}{y_{22}} B_2^* = 0 \implies \frac{B_1^*}{y_{12}} + \frac{B_2^*}{y_{22}} = s_2 - R_2^*$

Let $C_1 = s_1 - R_1^*$ and $C_2 = s_2 - R_2^*$. Solving this linear system for B_1^*:

$$B_1^* = \frac{y_{11} y_{12} (y_{21} C_1 - y_{22} C_2)}{y_{12} y_{21} - y_{11} y_{22}} \tag{132}$$

From the R_3 balance equation:

$$B_1^* - aR_3^* - \frac{a}{y_{33}} B_3^* = 0 \implies B_3^* = y_{33} \left(\frac{B_1^*}{a} - R_3^* \right) \tag{133}$$

From the R_5 balance equation:

$$a(xs_1 - R_5^*) - \frac{a}{y_{35}} B_3^* = 0 \implies R_5^* = xs_1 - \frac{B_3^*}{y_{35}} \tag{134}$$

Substituting B_3^*:

$$R_5^* = xs_1 - \frac{y_{33}}{y_{35}} \left(\frac{B_1^*}{a} - R_3^* \right) \tag{135}$$

By Liebig's Law of the Minimum, B_3 is limited by R_3 if the growth rate supported by R_3 is less than or equal to that supported by R_5:

$$\mu_{33} \frac{R_3^*}{R_3^* + K_{33}} \leq \mu_{35} \frac{R_5^*}{R_5^* + K_{35}} \tag{136}$$

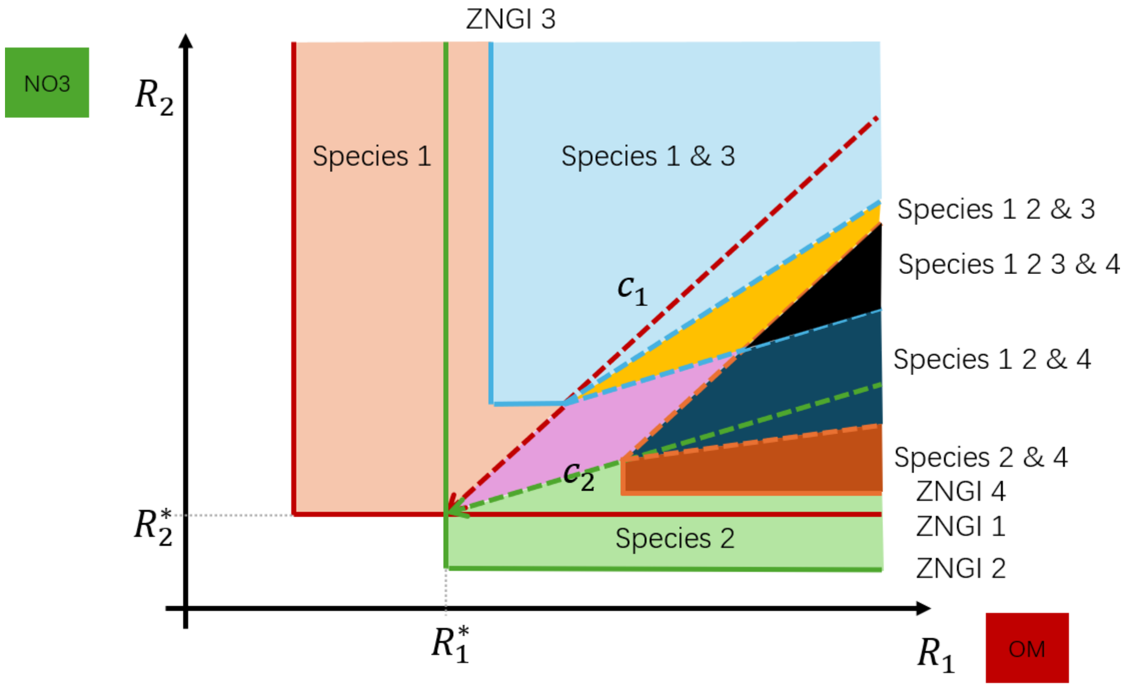

Figure 6: Graphical analysis of species 1, species 2, species 3, and species 4.

Since the left-hand side is equal to a at steady state, we have:

$$a \leq \mu_{35} \frac{R_5^*}{R_5^* + K_{35}} \implies R_5^* \geq \frac{aK_{35}}{\mu_{35} - a} \tag{137}$$

Let $R_{5,\min} = \frac{aK_{35}}{\mu_{35}-a}$. The condition for x is:

$$x s_1 - \frac{y_{33}}{y_{35}} \left(\frac{B_1^*}{a} - R_3^* \right) \geq R_{5,\min} \tag{138}$$

$$x \geq \frac{1}{s_1} \left[\frac{y_{33}}{y_{35}} \left(\frac{B_1^*}{a} - R_3^* \right) + R_{5,\min} \right] \tag{139}$$

The condition for x such that B_3 is limited by R_3 is:

$$x \geq \frac{1}{s_1} \left[\frac{y_{33}}{y_{35}} \left(\frac{y_{11} y_{12} (y_{21}(s_1 - R_1^*) - y_{22}(s_2 - R_2^*))}{a(y_{12} y_{21} - y_{11} y_{22})} - R_3^* \right) + R_{5,\min} \right] \tag{140}$$

where the steady-state substrate constants are:

$$R_1^* = \frac{aK_{21}}{\mu_{21} - a}, \quad R_2^* = \frac{aK_{12}}{\mu_{12} - a}, \quad R_3^* = \frac{aK_{33}}{\mu_{33} - a}, \quad R_{5,\min} = \frac{aK_{35}}{\mu_{35} - a}. \tag{141}$$

Note that for a linear growth kinetics, i.e., the growth of Liebig's Law follows $\min\{\mu_{11} R_1, \mu_{12} R_2\}$ instead of a Monod curve, the difference in the threshold only lies on the equilibrium concentration of substrates:

$$R_1^* = \frac{a}{\mu_{21}}, \quad R_2^* = \frac{a}{\mu_{12}}, \quad R_3^* = \frac{a}{\mu_{33}}, \quad R_{5,\min} = \frac{a}{\mu_{35}}. \tag{142}$$

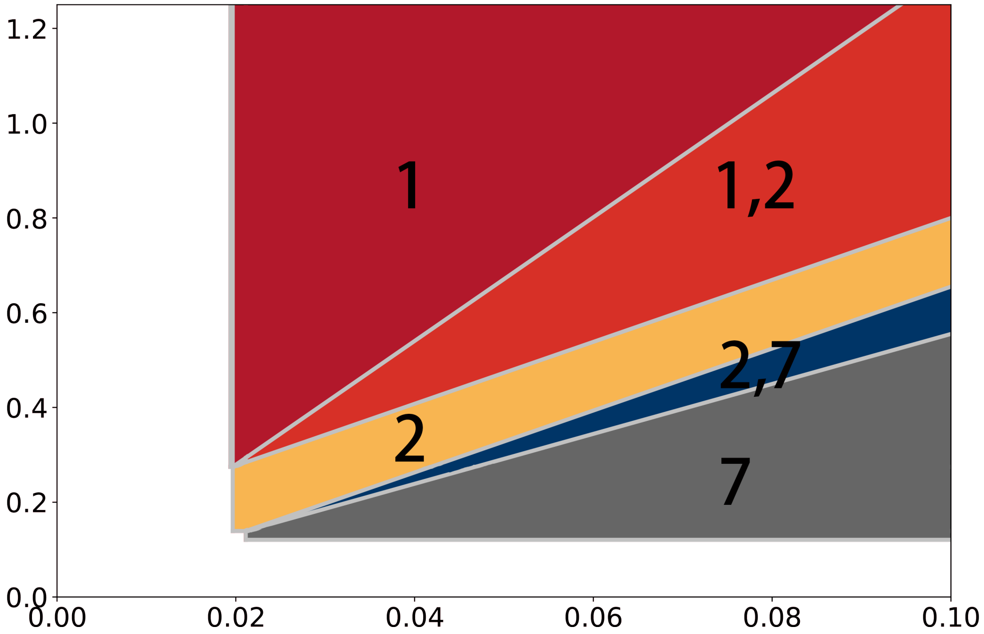

Figure 7: Graphical analysis of groups 1, 2 and 7.

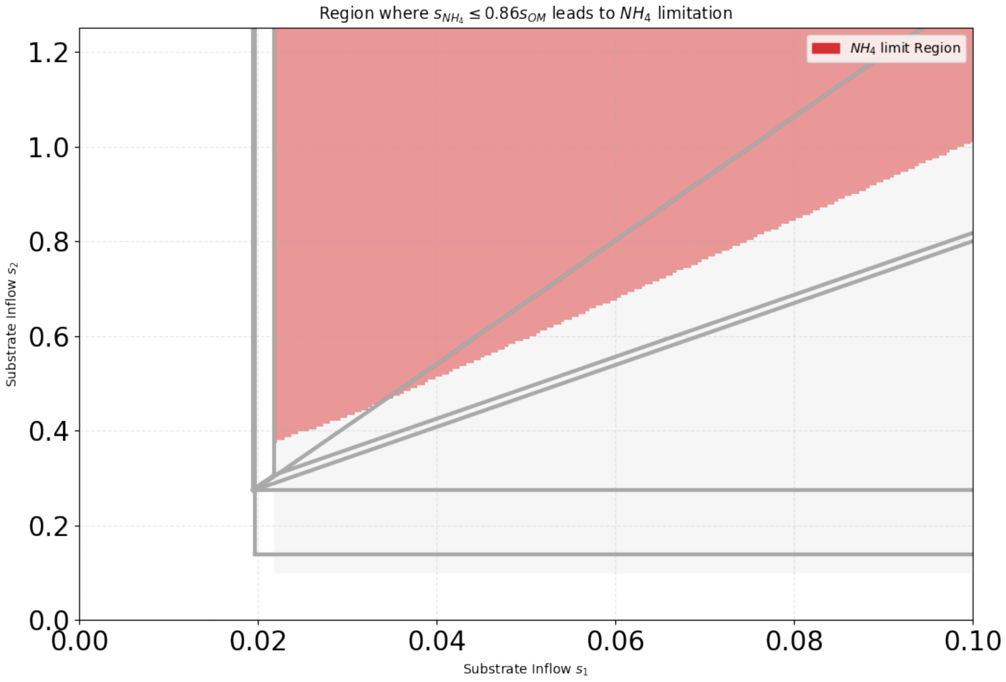

Figure 8: We assume the supply of ammonium is proportional to the OM remineralization in the range of 70-86%. In this range, annamox is most likely to be limited by ammonium, which promote coexistence with $NO_2 -> N_2$ denitrifiers.

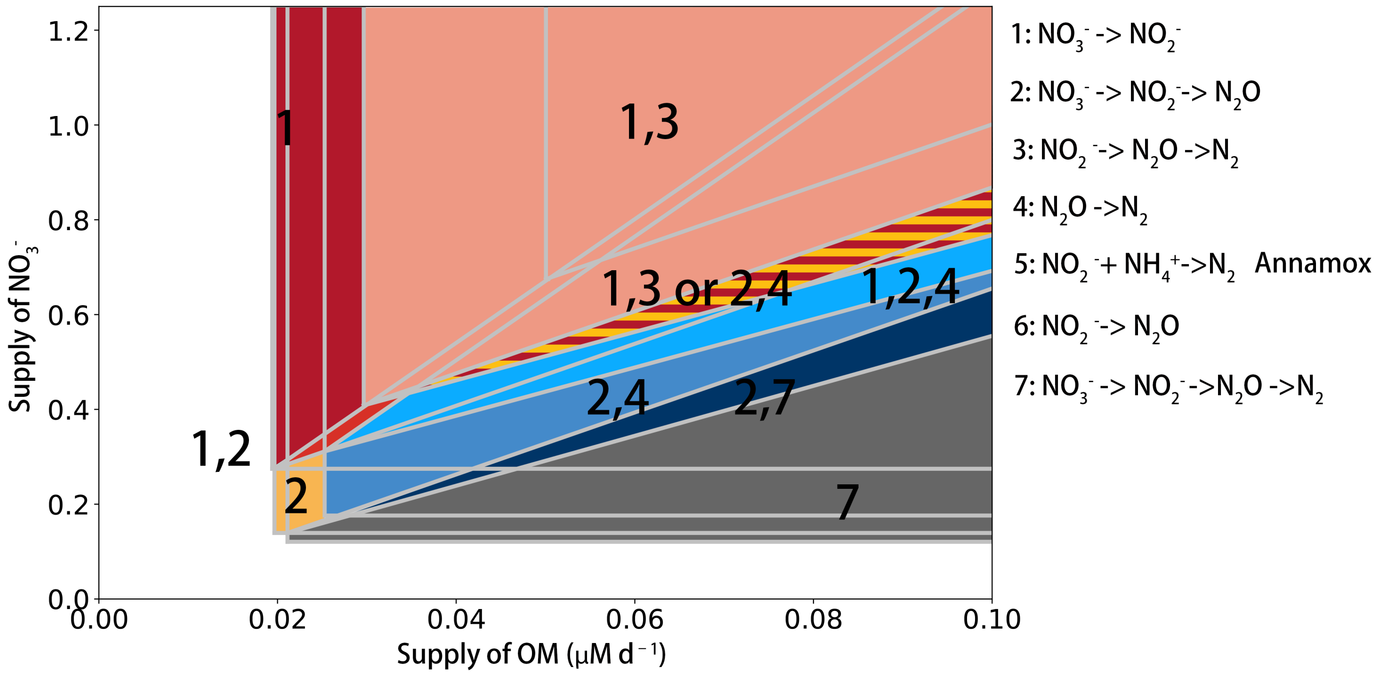

Figure 9: If anammox is a weaker competitor for NO_2 compared to NO_2 reducers, it will be completely excluded from the ecosystem.
